# Functionalized Membrane Domains: An Ancestral Feature of Archaea?

**DOI:** 10.3389/fmicb.2020.00526

**Published:** 2020-03-31

**Authors:** Maxime Tourte, Philippe Schaeffer, Vincent Grossi, Phil M. Oger

**Affiliations:** ^1^Université de Lyon, INSA Lyon, CNRS, MAP UMR 5240, Villeurbanne, France; ^2^Université de Strasbourg-CNRS, UMR 7177, Laboratoire de Biogéochimie Moléculaire, Strasbourg, France; ^3^Université de Lyon, ENS Lyon, CNRS, Laboratoire de Géologie de Lyon, UMR 5276, Villeurbanne, France

**Keywords:** Archaea, Thermococcales, membrane domains, archaeal lipids, membrane architecture, membrane function, evolution, adaptation

## Abstract

Bacteria and Eukarya organize their plasma membrane spatially into domains of distinct functions. Due to the uniqueness of their lipids, membrane functionalization in Archaea remains a debated area. A novel membrane ultrastructure predicts that monolayer and bilayer domains would be laterally segregated in the hyperthermophilic archaeon *Thermococcus barophilus*. With very different physico-chemical parameters of the mono- and bilayer, each domain type would thus allow the docking of different membrane proteins and express different biological functions in the membrane. To estimate the ubiquity of this putative membrane ultrastructure in and out of the order Thermococcales, we re-analyzed the core lipid composition of all the Thermococcales type species and collected all the literature data available for isolated archaea. We show that all species of Thermococcales synthesize a mixture of diether bilayer forming and tetraether monolayer forming lipids, in various ratio from 10 to 80% diether in *Pyrococcus horikoshii* and *Thermococcus gorgonarius*, respectively. Since the domain formation prediction rests only on the coexistence of di- and tetraether lipids, we show that all Thermococcales have the ability for domain formation, i.e., differential functionalization of their membrane. Extrapolating this view to the whole Archaea domain, we show that almost all archaea also have the ability to synthesize di- and tetraether lipids, which supports the view that functionalized membrane domains may be shared between all Archaea. Hence domain formation and membrane compartmentalization may have predated the separation of the three domains of life and be essential for the cell cycle.

## Introduction

Archaea are the main inhabitants of the harshest environments, regardless of whether the extreme conditions are temperature, salinity, hydrostatic pressure, pH, or scarce nutrients. This tolerance to extreme physical and chemical conditions has been associated with three specific characteristics of their membrane lipids, which greatly differ from their eukaryotic and bacterial counterparts (De Rosa et al., 1986a; Albers and Meyer, 2011): (1) archaeal ether bonds that are more chemically resistant than bacterial/eukaryal ester bonds, allow a tighter compaction of the lipids (Baba et al., 1999); (2) archaeal isoprenoid hydrocarbon chains that enhance membrane packing relative to linear acyl chains of bacterial/eukaryal lipids, create cell membranes with enhanced stability and impermeability (Komatsu and Chong, 1998); and (3) archaeal tetraether lipids can form monolayer membranes that are more rigid and impermeable than bacterial/eukaryal bilayer membranes classically made of diacylglycerols (Chong, 2010). The presence of bipolar tetraether lipids is supportive of hyperthermophilic (de Rosa et al., 1977) and acidophilic growth (Macalady et al., 2004) in Archaea. This view was further supported by the observation that the most extremophilic bacterium, *Thermotoga maritima* (*T*_max_ = 90°C), also produces tetraether membrane-spanning lipids (Sinninghe Damsté et al., 2007). All works to date demonstrate a strong correlation between the proportion of tetraether lipids and the adaptation to extreme acidity in thermoacidophiles (Boyd et al., 2013), and between the absence of tetraether lipids and the adaptation to extreme salinity or the adaptation to high pH (Kates, 1993). In contrast, no strong correlation have been highlighted for other extremes, nor for mesophilic conditions, which raises questions as to how archaea thriving in such habitats adapt to their specific lifestyles. This suggests that some environmental stress factors are not strong enough to drive measurable compositional shifts in membrane lipids or may involve adaptive routes not requiring an alteration of the diether/tetraether ratio, or that unknown alternative adaptive routes exist in Archaea.

Hence, the existence of neutrophilic hyperthermophiles, such as *Methanopyrus kandleri*, which is incapable of synthesizing tetraethers but grows optimally at 110°C (Hafenbradl et al., 1996), questions the need for these tetraether lipids for growth at high temperatures. Furthermore, a large number of hyperthermophilic archaea produce a mixture of tetra- and diether lipids while growing at temperatures near or above 100°C (Trincone et al., 1992). In these organisms, diether lipids often represent a large part of the membrane lipids, which implies that the membrane may contain domains of monolayer and domains of bilayer. Increasing temperature increases molecular motion in lipids. Hence, at high temperature, bilayers become more fluid, which impairs greatly their impermeability. In contrast, low temperatures will reduce molecular motion and make monolayers even more impermeable and less fluid. Thus, the stability of bi- and monolayer domains pose a challenge to either hyperthermophilic or mesophilic archaea. However, domains of divergent lipid composition allow one to envision that adaptation could involve the variation of physico-chemical properties intrinsic to these domains, i.e., the differential functionalization of the membrane. For instance, it could help explaining how the membrane of mesophilic archaea, which often contain a majority of tetraether lipids (Elling et al., 2017), remains functional, although the adaptive role for such lipids under mesophilic conditions remains questionable. Similarly, to explain the stability of *Thermococcus barophilus* bilayer domains under the hyperthermophilic conditions required for growth, it has been suggested that apolar polyisoprenoids sitting in the midplane of the membrane could extend the domain of stability to high temperatures (Cario et al., 2015; Salvador-Castell et al., 2020). All these hypotheses imply that the coexistence of domains of divergent lipid composition and physico-chemical behavior, e.g., monolayer and bilayer domains, could allow the anchoring of different proteins and confer these domains peculiar physiological and adaptive functions.

The formation of membrane domains in archaeal lipids has been directly observed in artificial mixtures of either tetraether (Bagatolli et al., 2000) or diether lipids (Salvador-Castell et al., 2020), and has been suggested to occur in natural mixtures of both diether and tetraether lipids to explain the extreme tolerance of the membrane of *T. barophilus (Cario et al., 2015)*. In the evolution of Archaea and archaeal membranes, we hypothesize that this particular adaptive route might have played a major role. To test this hypothesis, we looked at the ability of Archaea to harbor a differentially functionalized membrane, by focalizing on the diether/tetraether domain formation, which is the easiest to assess at the phylum level since it can be inferred from the sole analysis of membrane core lipid compositions. However, one bottleneck of this approach is the heterogeneous quality of the lipid data available in the literature. For instance, the reassessment of membrane lipid compositions using more adequate analytical approaches (Nishihara and Koga, 1991; Sugai et al., 2004; Cario et al., 2015) have highlighted strong discrepancies with those originally reported (De Rosa et al., 1986b, 1987; Marteinsson et al., 1999). To obtain a complete set of comparable membrane lipid compositions and constrain the level of trust we could have in published lipid compositions of Archaea, we reassessed the lipid compositions of the whole order Thermococcales, to which *T. barophilus* belongs.

We show here that most of the Thermococcales lipid compositions are significantly different from previously published work. In some instances, it confirmed the synthesis of previously undetected tetraether lipids, showing that all Thermococcales are able to synthesize both di- and tetraethers. These findings show that the membrane organization proposed for *T. barophilus* could be extended to the whole Thermococcales order and suggest that the ability to synthesize diethers and/or tetraethers could be inferred from taxonomically related species. We can thus propose the coexistence of monolayer and bilayer membrane domains to almost all contemporary archaeal orders with isolated representatives, with the exception of halophiles which possibly harbor only bilayers. Such a wide distribution supports the ability of the last archaeal common ancestor (LACA) to synthesize both diether and tetraethers lipids, and to harbor a membrane with functionalized domains.

## Materials and Methods

### Microorganisms and Growth Conditions

Information on the isolation location and optimal growth conditions of the 51 strains analyzed is displayed in Supplementary Table S1. Each strain was grown at pH 6.8 under anaerobiosis in a rich medium established for Thermococcales (TRM; Zeng et al., 2009) containing 3% NaCl and 10 g L^–1^ elemental sulfur, with the exception of *Thermococcus waiotapuensis* and *Thermococcus zilligii*, which were grown with 0.6% NaCl. *Pyrococcus* species were all grown at 98°C, whereas *Palaeococcus* and *Thermococcus* species were grown at 85°C, with the exception of *Thermococcus piezophilus* and *Thermococcus sibiricus*, which were grown at 75°C. Before growth, the medium was reduced by adding Na_2_S (0.1% final concentration). To confirm the assumptions based on our literature survey, additional strains were grown under their respective optimal conditions and included: *Aciduliprofundum boonei* DSM19572 (Reysenbach et al., 2006), *Aeropyrum pernix* DSM11879 (Sako et al., 1996), *Halobacterium salinarum* DSM3754 (Pfeiffer et al., 2019), *Haloferax volcanii* DSM3757 (Torreblanca et al., 1986), *Methanothermobacter thermautotrophicus* DSM1053 (Schönheit et al., 1980), *Methanocaldococcus jannaschii* DSM2661 (Jones et al., 1983), *Natronomonas pharaonis* DSM2160 (Tindall et al., 1984), *Pyrobaculum islandicum* DSM4184 (Huber et al., 1987), and *Sulfolobus acidocaldarius* DSM639 (Langworthy et al., 1974). Growth was monitored by counting with a Thoma cell, and growth curves were established for each species.

### Lipid Extraction and Analysis

Cultures (250 mL) in the late exponential phase were centrifuged (4000 × *g*, 45 min, 4°C) and rinsed twice with an isotonic solution. The cell pellets were then lyophilized overnight and kept at −80°C until cell hydrolysis, followed by lipid extraction. Lipid extraction was performed on three biological replicates for each strain.

**FIGURE 1:**
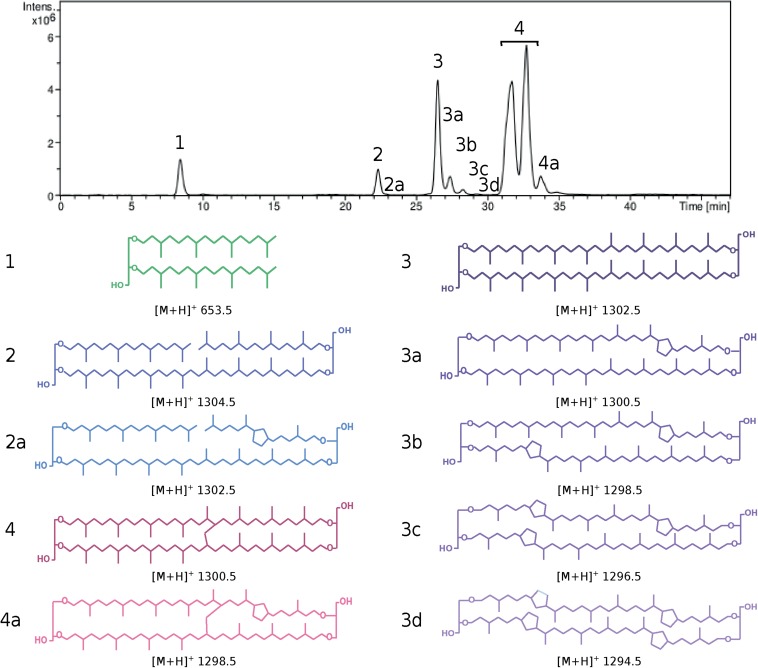
HPLC chromatogram **(top)** and lipid core structures **(bottom)** recovered from *Pyrococcus furiosus* after direct cell methanolysis (HCl/MeOH, 110°C). Cells were grown in TRM under optimal conditions. (1) Diphytanyl glycerol diether (DGD, green), (2 and 2a) glycerol trialkyl glycerol tetraethers with 0 (GTGT-0, dark blue) and 1 cyclopentane ring (GTGT-1, light blue), (3–3d) glycerol dibiphytanyl glycerol tetraethers with 0–4 rings (GDGT-0 to 4, dark to light purple) and (4 and 4a) glycerol monoalkyl glycerol tetraethers with 0 (GMGT-0, dark red) and 1 ring (GMGT-1, light red). [M+H]^+^: mass/charge ratio.

Previous studies of the lipid composition of solvent extracts from archaeal cells and environmental samples have illustrated possible analytical biases due to the selective extraction of some lipid classes over others (Huguet et al., 2010; Cario et al., 2015). To account for this disadvantage, lyophilized cells were directly hydrolyzed to remove polar head groups from the core lipids, which could then be quantitatively extracted (Cario et al., 2015). However, this procedure can also result in the removal of additional hydroxyl groups and the destruction of (poly)unsaturated lipids, and thus possibly reduce the retrieved lipid diversity. Following acid methanolysis of the dried cells (1.2 N HCl in methanol at 110°C for 3 h), core lipids were extracted by filtration over celite using methanol/dichloromethane (1:1, v/v). The resulting solvent extracts were dried under reduced pressure, solubilized in *n*-hexane/isopropanol or *n*-heptane/isopropanol (99:1, v/v) and analyzed by high-performance liquid chromatography (HPLC-MS) using an HP 1100 series LC-MS instrument equipped with an auto-injector and a Chemstation chromatography manager software. The analytical conditions were modified after Cario et al. (2015) *(Cario et al., 2015)*. Separations were achieved on a Prevail Cyano 3 microns column (150 mm × 2.1 mm; Grace Davison Discovery Sciences) maintained at 30°C. Injection volume was of 10 μl. Core lipids were eluted with a flow rate of 0.2 ml min^–1^, using the following gradient: 95% B (5 min isocratic) to 65% B in 30 min (5 min isocratic), then to 0% B in 1 min (10 min isocratic), and back to 95% B in 1 min (5 min isocratic), with A = 95% n-heptane : 5% isopropanol (v/v) and B = 100% n-heptane. Detection was achieved using an Esquire 3000+ ion trap mass spectrometer with an atmospheric pressure positive ion chemical ionization source (APCI-MS). Conditions for MS analyses were: nebulizer pressure 50 psi, APCI temperature 420°C, drying temperature 350°C, drying gas (N2) flow 5 L min^–1^, capillary voltage −2 kV, corona 4 μA, scan range m/z 600–2200. Under our analytical conditions, dialkyl glycerol diether with isoprenyl chains with 20 carbons (DGD) and its macrocyclic derivative, and diethers with one or two isoprenyl chains with 25 carbons were the only forms of diether lipids detected, whereas three tetraether structures were identified, namely glycerol dialkyl glycerol tetraethers (GDGT), glycerol trialkyl glycerol tetraethers (GTGT), and glycerol monoalkyl glycerol tetraethers (GMGT) (for lipid structures, refer to Figure 1 and Supplementary Figure S1). A standard solution containing core DGD and GDGT-0 in a 2/1 molar ratio highlighted a molar response factor of DGD *ca.* 10 times lower than that of GDGT-0. A response factor of 1 was assumed between DGD and the other diethers and between GDGT-0 and the other tetraethers. For each archaeal species, core lipid relative abundances were determined by integration of the peak area on the mass chromatograms corresponding to the [M+H]^+^ ion of the different core lipids, and the relative abundances of diethers were corrected by a factor of 10 relative to that of tetraethers.

### Statistical Analyses

Statistical analyses were computed in R (version 3.4.2; R Core Team, 2017). Principle component analysis (PCA) was conducted on the lipid proportions to determine the relative data dispersion for each strain. Due to the sparsity of the ring-containing core lipids in Thermococcales, the analysis was performed on the total proportions of each type of lipid skeleton, combining both cyclic and acyclic core structures, namely, DGD, GDGT, GMGT and GTGT (for lipid structures, refer to Figure 1). The Thermococcales average number of cyclopentane rings per lipid structure was represented by the RI calculated as follows (Schouten et al., 2007):

$$R\,I=\frac{\begin{array}{c} G\,D\,G\,T-1+G\,M\,G\,T-1+G\,T\,G\,T-1+2\times G\,D\,G\,T-\\ 2+3\times G\,D\,G\,T-3+4\times G\,D\,G\,T-4 \end{array}}{\begin{array}{c} G\,D\,G\,T-0+G\,M\,G\,T-0+G\,T\,G\,T-0+G\,D\,G\,T-1+G\,M\,G\,T-1+\\ G\,T\,G\,T-1+G\,D\,G\,T-2+G\,D\,G\,T-3+G\,D\,G\,T-4 \end{array}}$$

To correlate the environmental conditions and the core lipid compositions of Thermococcales, a classical canonical correlation analysis (CCA) was conducted using the lipid relative abundances and the physical and chemical properties of the hydrothermal vents from which each strain was isolated. The hydrothermal fluid composition was unavailable for 19 out of the 51 analyzed strains, and the CCA analysis was conducted based on 32 strains. Missing values for the 16 corresponding hydrothermal vents were estimated using the missMDA R package (Josse and Husson, 2016). Briefly, the package attributes a random, yet likely, value to the missing data based on similarities between individuals and links between variables. This value is then optimized through iterative PCA, until the algorithm reaches convergence. Data were not scaled prior to estimation, and the number of components used for estimating the missing values was set to 5, the optimal number of components determined by missMDA. This process resulted in positive estimated values, in agreement with the physical and chemical nature of the measured parameters (Supplementary Table S1). The variability in the missing value prediction was estimated using multiple imputations and the k-fold cross validation method implemented in missMDA (Josse and Husson, 2016). The prediction of the missing values of our dataset displayed a great variability across imputations, thus reducing the confidence in the estimated values, and therefore in the variables with initially missing values (i.e., Sr, Fe, Mn and H_2_S). The CCA was then performed using the CCA R package (González et al., 2008) on lipid composition (DGD, GDGT, GMGT, GTGT, and RI) and environmental condition matrices using the first two substantial canonical correlations.

## Results

### Thermococcales Exhibit a Vast Diversity of Core Structures

To estimate the possible ubiquity of the functionalized membrane domain organization within the Thermococcales order, the core lipid distribution of 51 strains of Thermococcales grown under optimal conditions was assessed by direct acid methanolysis followed by HPLC-APCI-MS. In contrast to previous reports, all Thermococcales species were able to produce both di- and tetraether lipids in our analysis (Table 1; for core lipid structures, refer to Figure 1). For instance, we showed that *P. kukulkanii* was able to produce *ca*. 66% of tetraether lipids and not 100% diethers. When both classes of molecules were already reported, our results often show drastic differences to data from the literature. The minimal lipid set, i.e., the core lipidome, contained DGD ([M+H]^+^ 653.5, compound 1, Figure 1), GTGT with no cyclopentane ring (GTGT-0, [M+H]^+^ 1302.5, compound 2, Figure 1) and GDGT with no cyclopentane ring (GDGT-0, [M+H]^+^ 1302.5, compound 3, Figure 1). In contrast, rarer core structures, namely, GMGT with no cyclopentane ring (GMGT-0, [M+H]^+^ 1300.5, compound 4, Figure 1) and ring-containing tetraethers (compounds 2a, 3a-d, and 4a, Figure 1), were detected in only 21 out of the 51 strains analyzed. All these core lipid structures were present simultaneously only in *Pyrococcus furiosus* and *Thermococcus* sp. P6 (Figure 1 and Table 1).

**TABLE 1:** Core lipid composition of the 51 Thermococcales strains.

	Diethers	Tetraethers								
	DGD	GDGT-0	GDGT-1	GDGT-2	GDGT-3	GDGT-4	GMGT-0	GMGT-1	GTGT-0	GTGT-1
*Palaeococcus ferrophilus*	42.9 ± 4.8	57.5 ± 4.8	ND	ND	ND	ND	ND	ND	0.5 ± 0.1	ND
*P. helgesonii*	26.0 ± 5.4	47.5 ± 8.6	ND	ND	ND	ND	26.4 ± 13.4	ND	0.2 ± 0.1	ND
*P. pacificus*	47.7 ± 5.8	51.3 ± 5.9	ND	ND	ND	ND	ND	ND	1.0 ± 0.1	ND
*Pyrococcus abyssi*	16.5 ± 1.4	83.4 ± 1.3	ND	ND	ND	ND	ND	ND	0.1 ± 0.2	ND
*P. “endeavori”* ES4	35.4 ± 0.4	64.5 ± 0.4	ND	ND	ND	ND	ND	ND	0.1 ± 0.0	ND
*P. furiosus*	39.3 ± 9.0	11.7 ± 2.8	2.4 ± 0.9	0.8 ± 0.3	0.2 ± 0.1	Traces	38.9 ± 4.8	4.4 ± 1.1	2.2 ± 0.9	0.1 ± 0.0
*P. glycovorans*	34.3 ± 13.1	65.3 ± 13.3	ND	ND	ND	ND	ND	ND	0.4 ± 0.2	ND
*P. horikoshii* OT3	9.1 ± 2.0	63.6 ± 3.8	0.6 ± 0.1	0.1 ± 0.1	ND	ND	28.7 ± 2.3	0.2 ± 0.1	ND	ND
*P. horikoshii* JA-1	8.2 ± 1.3	63.3 ± 4.6	5.1 ± 1.4	1.6 ± 0.4	0.1 ± 0.1	ND	20.9 ± 4.4	0.9 ± 0.6	0.1 ± 0.1	ND
*P. kukulkanii*	33.3 ± 10.5	65.9 ± 10.5	0.1 ± 0.1	ND	ND	ND	0.3 ± 0.3	ND	0.4 ± 0.1	ND
*P. woesei*	32.4 ± 14.8	65.4 ± 15.9	ND	ND	ND	ND	0.4 ± 0.2	ND	1.8 ± 1.1	ND
*Thermococcus acidaminovorans*	49.6 ± 4.5	49.0 ± 4.7	ND	ND	ND	ND	0.1 ± 0.1	ND	1.4 ± 0.2	ND
*T. aegaeus*	60.1 ± 11.1	36.8 ± 10.9	ND	ND	ND	ND	ND	ND	0.6 ± 0.2	ND
*T. aggregans*	57.2 ± 1.2	42.1 ± 1.6	ND	ND	ND	ND	ND	ND	0.7 ± 0.5	ND
*T. alcaliphilus*	61.5 ± 6.8	38.0 ± 6.7	ND	ND	ND	ND	ND	ND	0.4 ± 0.1	ND
*T. atlanticus*	30.3 ± 7.5	66.6 ± 6.4	ND	ND	ND	ND	2.5 ± 1.2	ND	0.6 ± 0.2	ND
*T. barophilus* MP	55.1 ± 8.0	44.3 ± 8.2	ND	ND	ND	ND	ND	ND	0.6 ± 0.2	ND
*T. barophilus* CH1	52.9 ± 10.1	44.5 ± 9.8	ND	ND	ND	ND	ND	ND	2.6 ± 0.2	ND
*T. barophilus* CH5	36.4 ± 11.1	61.9 ± 11.1	ND	ND	ND	ND	ND	ND	1.7 ± 0.1	ND
*T. barossii*	53.9 ± 1.4	45.2 ± 1.3	ND	ND	ND	ND	ND	ND	0.9 ± 0.1	ND
*T. celer*	60.1 ± 4.0	37.4 ± 3.7	ND	ND	ND	ND	2.3 ± 0.3	ND	0.2 ± 0.1	ND
*T. celericrescens*	69.00 ± 9.3	30.4 ± 9.1	ND	ND	ND	ND	ND	ND	0.6 ± 0.3	ND
*T. chitonophagus*	54.7 ± 4.7	43.9 ± 4.8	0.7 ± 0.1	0.3 ± 0.3	Traces	ND	ND	ND	0.4 ± 0.3	ND
*T. cleftensis*	60.9 ± 5.7	38.8 ± 5.7	ND	ND	ND	ND	ND	ND	0.4 ± 0.0	ND
*T. coalescens*	79.9 ± 1.5	19.9 ± 1.6	ND	ND	ND	ND	ND	ND	0.3 ± 0.2	ND
*T. fumicolans*	42.0 ± 2.6	57.9 ± 2.6	ND	ND	ND	ND	ND	ND	0.1 ± 0.1	ND
*T. gammatolerans*	55.8 ± 3.6	43.6 ± 3.6	ND	ND	ND	ND	ND	ND	0.5 ± 0.2	0.1 ± 0.1
*T. gorgonarius*	79.9 ± 5.8	19.8 ± 5.8	ND	ND	ND	ND	0.2 ± 0.1	ND	0.2 ± 0.1	ND
*T. guaymasensis*	51.7 ± 6.6	44.3 ± 6.0	ND	ND	ND	ND	3.4 ± 1.1	ND	0.6 ± 0.1	ND
*T. hydrothermalis*	56.6 ± 13.1	42.6 ± 12.9	ND	ND	ND	ND	ND	ND	0.8 ± 0.1	ND
*T. kodakarensis*	68.1 ± 8.0	31.5 ± 7.9	ND	ND	ND	ND	ND	ND	0.4 ± 0.1	ND
*T. litoralis*	62.4 ± 5.8	35.5 ± 5.2	1.0 ± 0.2	0.7 ± 0.1	0.1 ± 0.1	ND	ND	ND	0.4 ± 0.2	ND
*T. marinus*	70.9 ± 1.2	28.8 ± 1.2	0.1 ± 0.1	Traces	ND	ND	ND	ND	0.2 ± 0.0	ND
*T. nautili*	71.1 ± 4.5	28.6 ± 4.4	ND	ND	ND	ND	ND	ND	0.3 ± 0.3	ND
*T. onnurineus*	65.2 ± 4.2	33.1 ± 4.0	ND	ND	ND	ND	ND	ND	1.7 ± 0.4	ND
*T. pacificus*	59.1 ± 7.1	40.6 ± 6.9	ND	ND	ND	ND	ND	ND	0.3 ± 0.2	ND
*T. paralvinellae*	62.3 ± 6.3	35.7 ± 6.0	ND	ND	ND	ND	ND	ND	2.0 ± 0.3	ND
*T. peptonophilus*	59.2 ± 5.9	39.8 ± 6.2	ND	ND	ND	ND	0.6 ± 0.3	ND	0.5 ± 0.0	ND
*T. piezophilus*	65.7 ± 4.4	33.1 ± 4.2	ND	ND	ND	ND	ND	ND	1.2 ± 0.3	ND
*T. prieurii*	42.0 ± 2.5	57.5 ± 2.5	ND	ND	ND	ND	Traces	ND	0.5 ± 0.1	ND
*T. profundus*	39.1 ± 7.3	60.7 ± 7.1	ND	ND	ND	ND	0.1 ± 0.1	ND	0.1 ± 0.0	ND
*T. radiotolerans*	45.6 ± 13.2	51.5 ± 13.8	ND	ND	ND	ND	0.1 ± 0.1	ND	2.8 ± 0.7	ND
*T. sibiricus*	44.3 ± 2.9	55.5 ± 3.0	ND	ND	ND	ND	ND	ND	0.2 ± 0.1	ND
*T. siculi*	38.9 ± 6.1	59.9 ± 6.2	ND	ND	ND	ND	0.6 ± 0.2	ND	0.6 ± 0.2	ND
*T.* sp. AM4	59.1 ± 12.4	39.9 ± 12.1	ND	ND	ND	ND	ND	ND	1.0 ± 0.3	ND
*T.* sp. DT4	50.6 ± 2.8	47.8 ± 2.5	ND	ND	ND	ND	ND	ND	1.4 ± 0.2	0.2 ± 0.2
*T.* sp. P6	39.8 ± 6.7	55.9 ± 6.0	2.4 ± 0.5	1.1 ± 0.2	0.2 ± 0.00	Traces	0.5 ± 0.1	Traces	0.2 ± 0.0	Traces
*T. stetteri*	35.6 ± 5.7	63.3 ± 5.9	ND	ND	ND	ND	ND	ND	1.1 ± 0.3	ND
*T. thioreducens*	71.1 ± 6.2	28.1 ± 6.5	ND	ND	ND	ND	ND	ND	0.8 ± 0.3	ND
*T. waiotapuensis*	36.4 ± 8.1	22.1 ± 0.5	ND	ND	ND	ND	40.6 ± 8.2	ND	0.8 ± 0.0	ND
*T. zilligii*	42.5 ± 2.2	56.8 ± 2.3	ND	ND	ND	ND	ND	ND	0.7 ± 0.1	ND

*For each strain, values are the average of three biological replicates (relative % ± standard deviation). ND: not detected.*

### Environmental Parameters, Not Phylogeny, Drive Thermococcales Core Lipid Compositions

**FIGURE 2:**
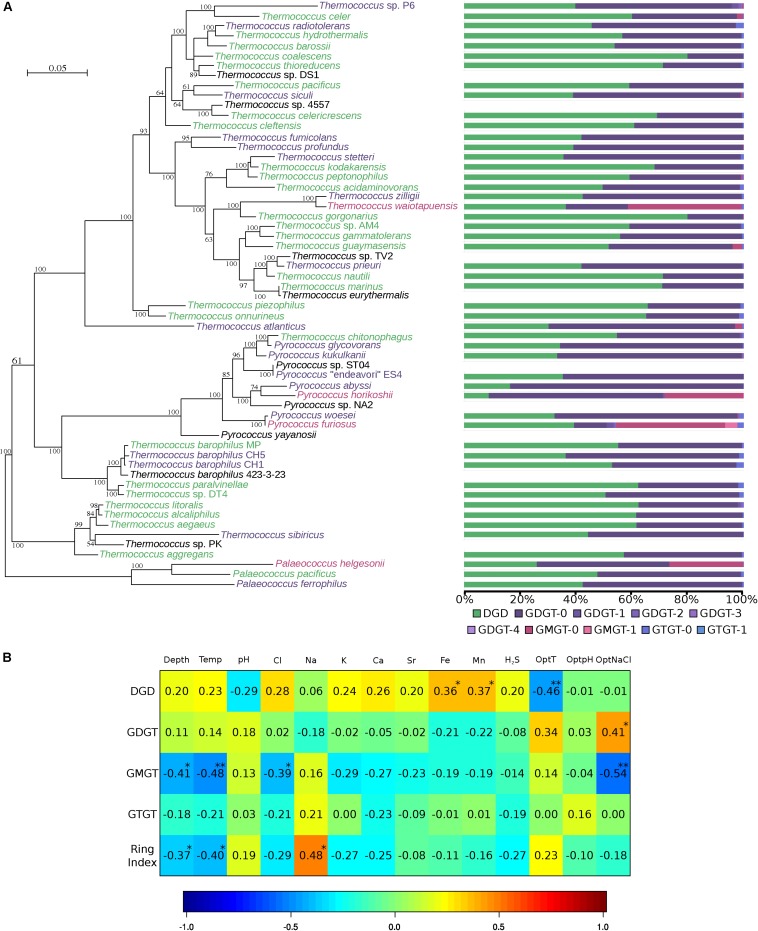
Thermococcales lipid composition is driven by environmental parameters, not by phylogeny. **(A)** Unrooted ML phylogeny of Thermococcales was inferred with the aligned concatenated 63 ribosomal proteins (9433 amino acid positions). The tree was inferred with PhyML (LG). The scale bar represents the average number of substitutions per site. Numbers at branches represent bootstrap values (1000 replicates, only values > 50% are shown). Core lipid relative proportions from Table 1 are represented as bar charts using the same color code as that in Figure 1. Lipid clusters are represented as the color of the species: green, DGD ≥ 50%; purple, GDGT ≥ 50%; red, GMGT ≥ 20%. For further details, see Supplementary Figure S1. **(B)** Correlation matrix between environmental parameters and lipid relative abundances. The correlations were calculated using the CCA R package (González et al., 2008). For further details, see Supplementary Table S1. Environmental data were available only for 32 strains isolated from 16 different hydrothermal vents. Missing ionic compositions were estimated using the missMDA R package (Josse and Husson, 2016). Proportions of ring-containing skeletons were added to their corresponding core structures, namely DGD, GDGT, GMGT, and GTGT, and the average ring number per core lipid structure was represented in the RI. *P*-values are represented as follows: 0.001 to 0.01 “**”, and 0.01 to 0.05 “*”.

Core lipid relative abundances in the Thermococcales order appeared to be strain-specific (Table 1 and Figure 2A). Thermococcales species displayed a great diversity of lipid content at the species level (Table 1 and Figure 2A). Often, the lipid dissimilarity among closely related strains even exceeded the between-species one, as exemplified by the three analyzed strains of *T. barophilus* (strain MP, 55% DGD, 44% GDGT-0 and 1% GTGT-0; strain CH1, 53% DGD, 45% GDGT-0 and 3% GTGT-0; and strain CH5, 36% DGD, 62% GDGT-0 and 2% GTGT-0); by the two species of *P. furiosus* (39% DGD, 12% GDGT-0 and 39% GMGT-0) and *P. woesei* (32% DGD, 65% GDGT-0 and 0.4% GMGT-0), which are almost identical at the genome level [sharing >99% identity over 100% of their genome (Kanoksilapatham et al., 2004)]; and by the closely related species *T. kodakarensis* (68% DGD and 32% GDGT-0) and *T. stetteri* (36% DGD and 63% GDGT). In contrast, distantly related species, such as *Thermococcus coalescens* and *Thermococcus gorgonarius* (*ca*. 80% DGD and 20% GDGT-0), or the two *Pyrococcus horikoshii* strains, OT3 (9% DGD, 64% GDGT and 29% GMGT) and JA-1 (8% DGD, 63% GDGT-0 and 21% GMGT-0), exhibited almost identical core lipid compositions. Similarly, no evidence of a genus-specific lipid composition was observed. Whereas *Thermococcus* species of the *T. barophilus* cluster, namely, *T. aegaeus*, *T*. *aggregans*, *T*. *alcaliphilus*, *T. litoralis*, *T. paralvinellae*, *T. sibiricus* and *T.* sp. DT4, had similar lipid compositions, with proportions of DGD ranging from 44% in *T. sibiricus* to 62% in *T. litoralis* (Table 1 and Figure 2A), and all *Pyrococcus* species appeared to share high proportions of tetraethers (Figure 2A), *Palaeococcus* and the remaining *Thermococcus* species displayed a great diversity of lipid compositions (Figure 2A). Furthermore, a clustering tree based on lipid compositions (Supplementary Figure S2) was neither congruent with the phylogenetic tree of Thermococcales based on concatenated ribosomal proteins (Figure 2A) or on whole-genome content (Supplementary Figure S4), nor with that of the concatenated lipid synthetic pathway genes (Supplementary Figure S3).

To test whether the core lipid composition of Thermococcales could be driven by environmental parameters, lipid proportions were correlated with the environmental conditions measured at the isolation site of each species and with optimal growth conditions in the laboratory (Figure 2B and Supplementary Table S1). The optimal growth temperature was negatively correlated (−0.46) with the DGD content, and positively correlated with the GDGT content (+0.34) and the average number of cyclopentane rings per molecule [i.e., the ring index, (RI)] (+0.23). Depth was positively correlated with DGD content (+0.20). The proportion of GMGT was negatively correlated with *in situ* temperature (−0.41) and was not significantly correlated with optimal growth temperature (−0.14). On the other hand, the GMGT composition was negatively correlated with the optimal growth salinity (-0.54). In contrast to all the other core structures detected in *Thermococcales*, the GTGT content was not significantly correlated with any of the environmental parameters considered (Figure 2B). Surprisingly, optimal and *in situ* pH conditions did not seem to be significantly correlated with any core lipid structure in Thermococcales (Figure 2B).

### Almost All Archaea Produce Both Di- and Tetraethers

**FIGURE 3:**
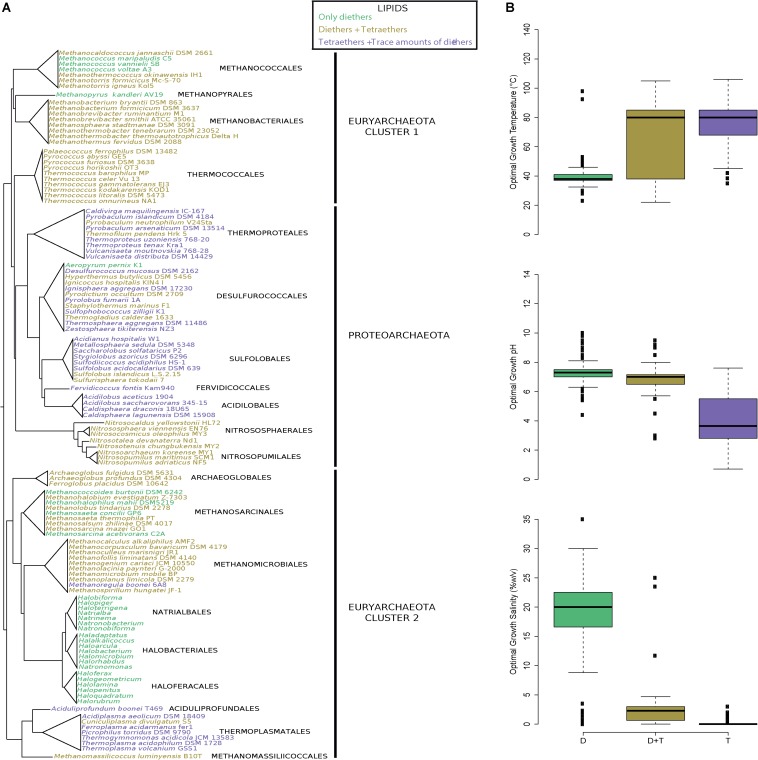
Plasma membranes containing both di- and tetraether lipids are widespread in Archaea. **(A)** Archaeal phylogeny and lipid composition. The tree topology has been adapted from Adam et al. (2017). **(B)** Box-and-whisker plots of the optimal growth conditions (temperature, pH and salinity) of all archaea with a known lipid composition (*n* = 440). Optimal growth conditions, lipid compositions and references are displayed in Supplementary Table S2. Species were colored according to their lipid compositions: only DGD (green), DGD and GDGT (dark yellow), and GDGT with trace amounts of DGD (purple).

To estimate the ubiquity of functionalized membrane domain organization, we compiled the core lipid distribution reported for 440 archaeal species belonging to all three archaeal kingdoms described by Petitjean et al. (2014), i.e., Euryarchaeota Clusters 1 and 2 and Proteoarchaota (Supplementary Table S2, Figure 3A). Unfortunately, 21 species belonging to 7 orders of Proteoarchaeota have never been screened for their diether content. Two scenarios account for an absence of diether lipids: (1) a recent loss of the ability to produce these lipids or (2) an analytical omission. In most cases, an analytical bias appears more parsimonious, as DGD was identified in one or more close relatives thriving in similar conditions. For instance, *Desulfurococcus amylolyticus* was screened only for its tetraether composition but was assumed to be able to synthetize diethers as its close relatives *D. mobilis* and *Ignicoccus hospitalis* produce trace amounts and 80.7% of DGD, respectively (Supplementary Table S2). Doing so, only the five Acidilobales species remained with an uncertain diether synthesis potential. However, several genomic studies have shown that *Acidilobus saccharovorans* possesses analogs of the genes required for diether lipid synthesis, i.e., short and long isopentenylphosphate synthases (IPPS) (Lombard et al., 2012), geranylgeranylglycerolphosphate synthase (GGGPS) (Villanueva et al., 2014; Coleman et al., 2019) and digeranylgeranylglycerolphosphate synthase (DGGGPS) (Villanueva et al., 2014), which argues in favor of its ability to produce diether lipids. GGGPS, DGGGPS and IPPS are key enzymes in the archaeal membrane lipid biosynthetic pathway. Indeed, short-chain IPPS is involved in the condensation of isopentenyl diphosphate and dimethylallyl diphosphate to form polyisoprenoids up to 25 carbon (Tachibana et al., 2000), such as geranylgeranyl diphosphate (GGPP, 20 carbon-long), whereas long-chain IPPS catalyzes the reaction for longer chains (Hemmi et al., 2002). GGGPS catalyzes the condensation of one GGPP onto glycerol-1-phosphate (G1P) (Zhang and Poulter, 1993b; Nemoto et al., 2003) and DGGGPS catalyzes the condensation of the second GGPP on the remaining hydroxyl group of G1P (Zhang and Poulter, 1993a; Hemmi et al., 2004). These enzymes eventually end on forming a diether lipid intermediate for the ezymes involved in polar headgroup addition, and such a biosynthetic pathway has been experimentally proven (Zhang and Poulter, 1993a). To date, there is no evidence that GGGPS and DGGGPS can accept polyisoprenoid chains longer than 20 or 25 for a few of them. Thus, it remains very unlikely that the GGGPS and DGGGPS homologues of Acidolobales could lead to the synthesis of tetraethers via the addition of C40-polyisoprenoids onto G1P. Thus, it is very probable that the presence of the IPPS, GGGPS and DGGPS genes in Acidolobales indicates that these archaea have the ability to synthesize diethers, but is not telling the length of their side chains, C20 or C25. It is thus reasonable to assume that all the Acidilobales would share this ability. In addition, we also highlighted an underestimation of the diether content of *A. boonei*, *P. islandicum*, and *S. acidocaldarius*, three species initially described as synthesizing only trace amounts of DGD (Supplementary Table S2). Indeed, using our procedure, they respectively exhibited 1.9, 5.3, and 11.2% of DGD, respectively (Supplementary Figure S1 and Supplementary Table S3).

Our lipid survey revealed that diether lipids are produced in all species of archaea (except the 21 Proteoarchaeota species in which this remains putative), regardless of whether they are acidophilic (e.g., *Thermoplasma acidophilum* and *S. acidocaldarius*), methanogenic (e.g., *Methanomassilicoccus lumyniensis* and *M. kandleri*), hyperthermophilic (e.g., *Pyrolobus fumarii* and *Archaeoglobus fulgidus*), halophilic (e.g., *H. salinarum* and *H. volcanii*), or mesophilic species (e.g., *Nitrososphaera viennensis* and *Nitrosopumilus maritimus*) (Figure 3A and Supplementary Table S2) (Adam et al., 2017). Diether abundances nonetheless varied from one species to another, with proportions ranging from trace amounts in acidophiles to 100% in halophiles (Supplementary Table S2). Similarly, this survey of the core lipid composition throughout the entire archaeal domain showed that both di- and tetraether lipids are present in all major lineages (185 species, 19 orders), the notable few exceptions being the hyperthermophile *A. pernix* and halophilic (248 species, 3 orders) and some methanogenic species, i.e., Methanococcoides (2 species), Methanococcus (3 species) and Methanohalophilus (1 species) which harbor only diether lipids (Figure 3 and Supplementary Table S2). However, as aforementioned, extraction methods often lead to biased lipid compositions which may specifically overlook tetraether lipids. For instance, only diethers were identified during the first descriptions of *T. barophilus*, *T. celer*, and *P. kukulkanii* (De Rosa et al., 1987; Marteinsson et al., 1999; Callac et al., 2016). Thus, using the same parsimony approach we applied to estimate the diether synthesis ability of the few uncertain Proteoarchaeota species, we considered that if one or more species of a genus possesses the ability to synthesize tetraethers lipids, then all members of the genus share this ability. For instance, only diether lipids were identified in *Methanococcus aeolicus*, *Methanococcus maripaludis*, and *Methanococcus voltae*, but we assumed these species to be able to synthesize tetraethers because *Methanococcus vannieli* and all the other Methanococcales do so. We further confirmed the methanogen’s ability to produce tetraether lipids by identifying such compounds in the core lipid extracts of *M. jannaschii* and *M. thermautotrohicus* (Supplementary Figure S1 and Supplementary Table S3). Overall, only halophilic archaea and *A. pernix* appeared to synthetize diether lipids only. This assumption was further confirmed by the absence of tetraethers in the core lipid extracts of *H. salinarum*, *H. volcanii*, *N. pharaonis*, and *A. pernix* (Supplementary Figure S1 and Supplementary Table S3).

Similarly to Thermococcales, all archaeal core lipid compositions appeared correlated with their respective optimal growth conditions. Indeed, the species producing a large majority of tetraethers tended to be thermoacidophiles (median optimal growth conditions: 80°C, pH 3.6, 0% NaCl), whereas those producing both di- and tetraether lipids were mainly thermoneutrophiles (median optimal growth conditions: 80°C, pH 7.0, 2.3% NaCl) (Figure 3B). Species harboring a membrane composed of solely diether lipids were halophiles (median optimal growth conditions: 38°C, pH 7.3, 20.0% NaCl), with the exception of the aforementioned methanogenic and hyperthermophilic species (Figure 3B).

## Discussion

### A Common Functional Membrane Architecture Emerges From the *Thermococcales* Panlipidome

We investigated the core lipid composition of all available strains of Thermococcales to estimate the confidence we could have in the data originally reported in the literature. Our data confirm that all the Thermococcales have the ability to synthesize both diether and tetraether lipids and that some lipid compositions previously reported might be erroneous. Our results further highlighted a greater core structure diversity than previously described in Thermococcales, namely DGD, GDGT, GTGT, GMGT and their ring-containing derivatives (Figure 1). Considering the diversity of the polar head groups already identified in Thermococcales, e.g., phosphatidyl(poly)hexoses, phosphatidylhexosamines, phosphatidylglycerol, and phosphatidic acid (Sprott et al., 1997; Marteinsson et al., 1999; Meador et al., 2014), together with the existence of isoprenoid core structures with additional methyl groups (Meador et al., 2014; Bauersachs et al., 2015) and of unsaturated hydrocarbons (Lattuati et al., 1998; Cario et al., 2015), we estimate the Thermococcales panlipidome to contain more than 100 distinct membrane lipid structures. Similarly, diverse lipid compositions were previously reported for other archaeal orders, e.g., Sulfolobales (Koga and Morii, 2005), Nitrosopumilales (Elling et al., 2017) and Methanosarcinales (Koga et al., 1998). Understanding the roles of such lipid diversity and how they organize into a functional, adaptable membrane is now of critical importance in the field of archaeal cell biology.

With the novel membrane organization into functionalized domains suggested for *T. barophilus* MP, based on the presence of both di- and tetraether lipids (Cario et al., 2015), the present set of data indicates that the ability to organize the membrane into separate domains may be shared between all Thermococcales. Similar to eukaryotic and bacterial membrane heterogeneities (Lingwood and Simons, 2010; López and Kolter, 2010), distinct physiological functions could be supported by separate bilayered and monolayered membrane domains. Indeed, due to the structural differences in their hydrophobic cores, diethers and tetraethers may anchor distinct membrane protein populations. For instance, the reduced lateral motion of tetraethers renders monolayered domains less susceptible to motion and deformation than bilayered domains (Shinoda et al., 2005), which can enhance the anchoring of proteins less prone to relocation, e.g., heavy complexes such as the archaellum and the S-layer (Albers and Jarrell, 2015; Rodrigues-Oliveira et al., 2017). In contrast, the more flexible bilayer domains may be prone to more dynamic processes, such as nanotube and vesicle formation (Marguet et al., 2013) which is favored by the presence of apolar hydrocarbons within the membrane midplane (Salvador-Castell et al., 2020). Hence, similar to that in Eukarya and Bacteria, if the archaeal membrane does contain separate domains with distinct lipid compositions, these domains should support differential functions by hosting distinct proteins.

### Lipid Distributions Highlight Specific Adaptive Functions

Our results demonstrate that the core lipid composition of Thermococcales species does not reflect their phylogenetic relationships, as already reported for other archaeal lineages, such as methanogenic and halophilic archaea (Koga et al., 1998; Kamekura and Kates, 1999). In contrast, the different core lipid structures detected in Thermococcales correlated with growth parameters (Figure 2B), suggesting that they might be involved in the adaptive response to these parameters. Indeed, the positive correlations observed between optimal growth temperature and GDGT and RI are in agreement with the initial proposal that GDGT participate in the adaptation to high temperatures (De Rosa et al., 1986a) and that the presence of cyclopentane rings further increases the compaction, rigidity and impermeability of the membrane (Gabriel and Chong, 2000; Chong, 2010). The positive correlation between DGD and the isolation depth further supports the role of diethers in adaptation to HHP (Kaneshiro and Clark, 1995; Cario et al., 2015). Indeed, HHP has been demonstrated to rigidify plasma membranes, decreasing their fluidity and permeability (Kato and Hayashi, 1999; Winter and Jeworrek, 2009), and diether lipids tend to exert the opposite effect on cell membranes. GMGT have been only recently discovered in a limited number of archaeal species, now including 21 out of the 51 Thermococcales species tested (Table 1). They have been tentatively associated with (hyper)thermophily (Morii et al., 1998; Sollich et al., 2017) because of the relationship observed between the GMGT proportion in membranes and the optimal growth temperature of *Ignisphaera aggregans* (39%, *T*_opt_ = 93°C), *Methanothermus fervidus* (25%, *T*_opt_ = 83°C), and *Methanothermobacter thermautotrophicus* (0.4%, T_opt_ = 65°C) (Knappy et al., 2011). However, our results do not support such an adaptive role, as the proportion of GMGT was neither positively correlated with *in situ* nor optimal growth temperatures (Figure 2B). On the other hand, the negative correlation between GMGT and optimal growth salinity points toward a possible role of these core lipid structures in adaptation to low salinity (Figure 2B). Indeed, the presence of an intramolecular C-C bond may limit the independent motions of the two alkyl chains of the tetraethers in addition to crowding the membrane internal space, enhancing membrane packing and creating an effective barrier to water and ion fluxes. Such an effect has been demonstrated for macrocyclic diether lipids, which have a structure resembling that of GMGT, with a C-C bond linking the two phytanyl chains (Dannenmuller et al., 2000). The relative abundance of GMGT (41%) in *T. waiotapuensis*, one of the few strains isolated from a terrestrial hot spring, suggests that these core structures may have played a critical role in the transition from marine hydrothermal vents to continental freshwater hydrothermal systems. Deciphering the lipid composition of freshwater-adapted Thermococcales may help in resolving the adaptive functions of these very particular core lipid structures. In contrast to GMGT, GTGT were detected in small proportions in all 51 Thermococcales strains, with a relative abundance ranging from trace amounts in *T. profundus* to 2.6% in *T. radiotolerans* (Table 1), and in many other archaeal species (Supplementary Table S2). Due to their peculiar structure gathering two C_20_ and one C_40_ isoprenoid chains (Figure 1), GTGT might support very original adaptive functions. However, molecular dynamics simulations showed that the physical and chemical properties of a membrane constituted of either GTGT-0 or GDGT-0 are highly similar (Shinoda et al., 2005). Thus, considering that they did not correlate with any of the parameters examined (Figure 2B), GTGT might not play an adaptive role in Thermococcales or other archaeal lineages but could rather be intermediates in the biosynthesis of tetraethers, as previously suggested (Koga and Morii, 2007). Further studies are required to fully understand the biological relevance of the various archaeal core lipid structures.

### Lipid Compositions Are Consistent With the Head-to-Head Condensation Tetraether Lipid Biosynthetic Pathway

Although the route to archaeal diether lipids from acetyl-CoA is now well characterized (Koga and Morii, 2007), the biosynthetic pathway leading to tetraethers remains elusive. Two putative pathways have been suggested so far: (1) tetraether lipids could result from the head-to-head condensation of two preexisting DGD (Nemoto et al., 2003; Koga and Morii, 2007), or (2) complete C_40_ alkyl chains could be fully synthesized prior to their condensation with two glycerol moieties (Villanueva et al., 2014).

If tetraether lipids were synthesized *via* the head-to-head condensation pathway proposed by Koga and Morii (2007), one would expect to detect traces of GTGT and DGD as intermediates of GDGT synthesis in strains which membranes are composed of tetraether lipids. In contrast, if tetraethers were synthesized via the C_40_ alkyl pathway proposed by Villanueva et al. (2014), one would expect to systematically detect diether lipids with either two C_40_ alkyl chains or with a mix of C_20_ and C_40_ alkyl chains as intermediates, which is not the case. The low abundance of DGD and GTGT in acidophiles rather supports their transient state as tetraether precursors, an hypothesis that has already been suggested by isotope probing studies of various archaea (Langworthy, 1982; Nishihara et al., 1989; Kellermann et al., 2016). Moreover, the presence of a small proportion of GTGT in every Thermococcales species investigated (Table 1) together with their apparent lack of adaptive role is coherent with a putative function as biosynthetic intermediates between diether and tetraether lipids (Figure 2B). The head-to-head condensation pathway is therefore favored over the one-shot synthesis of the C_40_ alkyl chains. GTGT resulting from the condensation of one alkyl chain of two DGD molecules would thus constitute the very first form of tetraethers synthesized in the cell. The condensation of the second alkyl chain would subsequently lead to the final GDGT. Remarkably, in our analyses, GMGT and ring-containing GDGT tended to be produced within the same species (e.g., *P. furiosus*, *T*. sp. P6 and *P. horikoshii*) in response to similar environmental conditions, which negatively mirrors the correlations observed for acyclic GDGT (Table 1 and Figure 2B). These results confirm a suggested link between GDGT and ring-containing GDGT and GMGT (De Rosa et al., 1986a; Schouten et al., 2008). Hence, all the lipid data available to date support a pathway for tetraether lipid biosynthesis from diether lipid precursors.

### Compositionally Differentiated Membrane Domains Might Be a Universal Feature of Archaea

DGD, GDGT-0, and GTGT-0 were detected in all Thermococcales strains investigated (Table 1). Considering GTGT as intermediates in the biosynthesis of tetraethers and their minor proportions, one can say that the Thermococcales plasma membrane is composed of DGD and GDGT-0. Based on the diether/tetraether lipid ratio of the most basal branches of each Thermococcales phylogenetic group, e.g., *P. ferrophilus*, *T. aggregans*, *T.* sp. DT4, *P. furiosus*, *T. atlanticus*, *T. onnurineus*, *T. marinus*, and *T. cleftensis*, the proportions of DGD and GDGT in the last Thermococcales common ancestor (LTCA) can be estimated to be *ca*. 50% each (Figure 2A). These results suggest that the LTCA may have harbored a membrane organization similar to that of *T. barophilus*, e.g., a differentiated domain-containing membrane. The similarities within Thermococcales regarding natural environments, genomic contents and now lipid compositions all converge to suggest that the LTCA thrived in a deep marine hydrothermal vent, similar to most of the current Thermococcales species (Supplementary Table S1). Peculiar core lipid distributions may then have evolved from this ancestral composition (*ca.* 50% of both diether and tetraether lipids) to fit with different environmental conditions, e.g., the production of GMGT for freshwater hydrothermal vents. Similarly, our survey of lipid composition throughout the entire archaeal domain unveiled diethers and tetraethers in almost every archaea investigated (Figure 3A). Thus, the proposed domain-containing membrane organization may be a widespread feature not only in Thermococcales but also within the whole archaeal domain, with the notable exception of halophiles and *A. pernix*. As for the archaeal root representing the LACA, which is supposed to lie between the Euryarchaeota Cluster 2 and Cluster 1/Proteoarchaeota groups (Raymann et al., 2015), the present archaeal lipid data set suggests that the LACA may have harbored a membrane containing both di- and tetraether lipids and, thus, could already have contained functionalized membrane domains, as proposed for *T. barophilus* MP (Cario et al., 2015).

Our analysis confirms a dual role for tetraethers in the adaptation to both high temperature and acidic conditions, as already proposed (Macalady et al., 2004; Boyd et al., 2013). Hence, the putative lipid composition of the LACA, made of both di- and tetraether lipids, would suggest that it has thrived in a near-neutral pH, thermophilic environment, which is in good agreement with the most recent evolutionary scenarios (Groussin and Gouy, 2011). From this ancestral lipid composition, three types of lipid composition matching distinct environmental settings would have been selected: (1) equimolar amounts of di- and tetraether lipids in response to mesophilic and thermophilic environments at near-neutral pH, e.g., Nitrosopumilales, Methanomicrobiales, and Thermococcales; (2) loss or drastic reduction of the tetraether lipid synthesis under hypersaline conditions, e.g., all haloarchaea; and (3) drastic increase in tetraether lipid synthesis in acidic environments, e.g., Sulfolobales and Thermoplasmatales (Figure 3).

Archaeal lipid compositions showed that all archaea produce diethers, whereas none of them seems able to produce only tetraethers (Figure 3A and Supplementary Table S2). Thus, diethers appear to be the most likely ancestral form of archaeal core lipids, which is congruent with a recent investigation of lipid biosynthetic genes that traced the archaeal diether origin back to the last universal common ancestor (LUCA) (Coleman et al., 2019). This result is also in agreement with the head-to-head condensation hypothesis for tetraether biosynthesis (Koga and Morii, 2007), which requires preexisting diether lipids. The presence of di- and tetraether lipids in LACA would imply that tetraethers and functionalized membrane domains predated LACA and may have already been present in LUCA. Alongside the existence of functionalized membrane domains in bacterial and eukaryotic membranes (Lingwood and Simons, 2010; López and Kolter, 2010), the functionalized domain-containing membrane of archaea would trace this feature deep into evolutionary history, suggesting that membrane domains are an ancient feature of cellular life and a fundamental membrane organizing principle.

## Conclusion

Resolving the core panlipidome of the hyperthermophiles *Thermococcales* highlighted the ability of all species to produce both diether and tetraether lipids in significant amounts, allowing extension of the functionalized-domain membrane organization hypothesis to the whole lineage. Similarly, with the possible exception of haloarchaea and a few methanogens, most known archaeal lineages are able to synthesize both di- and tetraether lipids, suggesting that the functionalized-domain membrane model might be valid for (almost) all archaea and, more importantly, for the LACA. In this view, the evolutionary scenario for archaeal membranes proposes that membrane domains are essential for membrane function and that they potentially originate back from the cenancestor, while specific lipid compositions reflect specific adaptations to environmental variables. In this study, we could only consider the role of the hydrophobic moiety in the evolution of the membrane. Several studies with bacterial or eukaryal lipids have shown that the polar headgroups also play an important role in the physico-chemical behavior of the membrane (physical parameter values, specific affinity for membrane proteins, etc.). Thus, it is likely that polar headgroups play a similar central role in the function and the stability of the archaeal membrane. For instance, the lipids of *Picrophilus oshimae*, an extreme acidophile, cannot self-assemble under neutral pH and require pH values lower than 4 to do so (Van de Vossenberg et al., 1998). Further elucidation of the role of these polar headgroups on membrane physico-chemical properties may prove essential to understand the lateral organization and the adaptation to the different environmental variables. For example, it may help to understand how Archaea with similar core lipid composition can be adapted either to low or high temperatures.

## Data Availability Statement

All datasets for this study are included in the article/Supplementary Material.

## Author Contributions

PO, PS, and VG were responsible for the conceptualization, funding acquisition, and methodology. MT worked on the formal analysis, the visualization and the writing of the original draft. MT and PS carried out the investigation. MT, PO, PS, and VG were responsible for the project administration, reviewing and editing the manuscript. PO supervised the study. MT, PO, and PS validated the study.

## Conflict of Interest

The authors declare that the research was conducted in the absence of any commercial or financial relationships that could be construed as a potential conflict of interest.
