# Supplementary material for: Functionalized Membrane Domains: An Ancestral Feature of Archaea?
Source: Front Microbiol. 2020 Mar 31;11:526. doi: 10.3389/fmicb.2020.00526 (PMC7137397; doi:10.3389/fmicb.2020.00526)
Supplement: Supplementary file 1 [file Data_Sheet_1.PDF]

Supplementary Material

1.1 Supplementary Figures

Figure S1. HPLC chromatograms (top) and lipid core structures (bottom) identified in *Halobacterium salinarum*, *Haloferax volcanii*, *Natronomonas pharaonis*, *Aeropyrum pernix*, *Methanocaldococcus jannaschii*, *Methanothermobacter thermautotrophicus*, *Aciduliprofundum boonei*, *Pyrobaculum islandicum* and *Sulfolobus acidocaldarius*. Cells were grown under their respective optimal conditions as described in the literature. (1) Diphytanyl glycerol diether (DGD, green), (1a) Macrocyclic DGD (M-DGD, light green), (1') 2-O-phytanyl-3-O-sesterterpanyl-sn-glycerol and/or 2-O-sesterterpanyl-3-O-phytanyl-sn-glycerol diether (C25, light yellow), (1'') disesterterpanyl glycerol diether (diC25, dark yellow), (2) glycerol trialkyl glycerol tetraethers with no cyclopentane ring (GTGT-0, blue), (3 to 3f) glycerol dibiphytanyl glycerol tetraethers with 0 to 6 rings (GDGT-0 to -6, dark to light purple) and (4 and 4a) glycerol monoalkyl glycerol tetraethers with 0 (GMGT-0, dark red) and 1 ring (GMGT-1, light red). $[M+H^{+}]$: mass/charge ratio.

Figure S2. Thermococcales core lipid composition clustering. A hierarchical clustering on principal components was conducted with the FactoMineR(Lê et al., 2008) R package (version 1.38) using Euclidean distance and the average linkage method on the mean core lipid abundances without prior data scaling. The optimal number of clusters was automatically computed using the highest relative loss of inertia.

Figure S3. Thermococcales core lipid composition vs. lipid synthesis gene phylogeny. Unrooted ML phylogeny of Thermococcales was inferred from the aligned concatenated protein sequences of the five core genes of archaeal lipid synthesis, e.g. the CDR archaeaol synthase (TERMP_01811), the (S)-3-O-geranylgeranylglyceryl phosphate synthase (TERMP_01020), (S)-2,3-di-O-geranylgeranylglyceryl phosphate synthase (TERMP_02000), the Digeranylgeranylglyceryl phospholipid reductase (TERMP_00654) and the multifunctional geranyl diphosphate/farnesyl diphosphate/geranylgeranyl diphosphate synthase (TERMP_00147) (1,467 amino acid positions). The tree was inferred with PhyML (LG). The scale bar represents the average number of substitutions per site. Numbers at branches represent bootstrap values (1000 replicates, values > 50% are shown). Core lipid relative proportions from Table 1 are represented as bar charts using the same color code as that in Figure 1.

Figure S4. **Thermococcales core lipid composition vs. whole genome phylogeny.** Unrooted phylogeny of Thermococcales was inferred from whole genomes suing REALPHY. The scale bar represents the average number of substitutions per site. Core lipid relative proportions from Table 1 are represented as bar charts using the same color code as that in Figure 1.

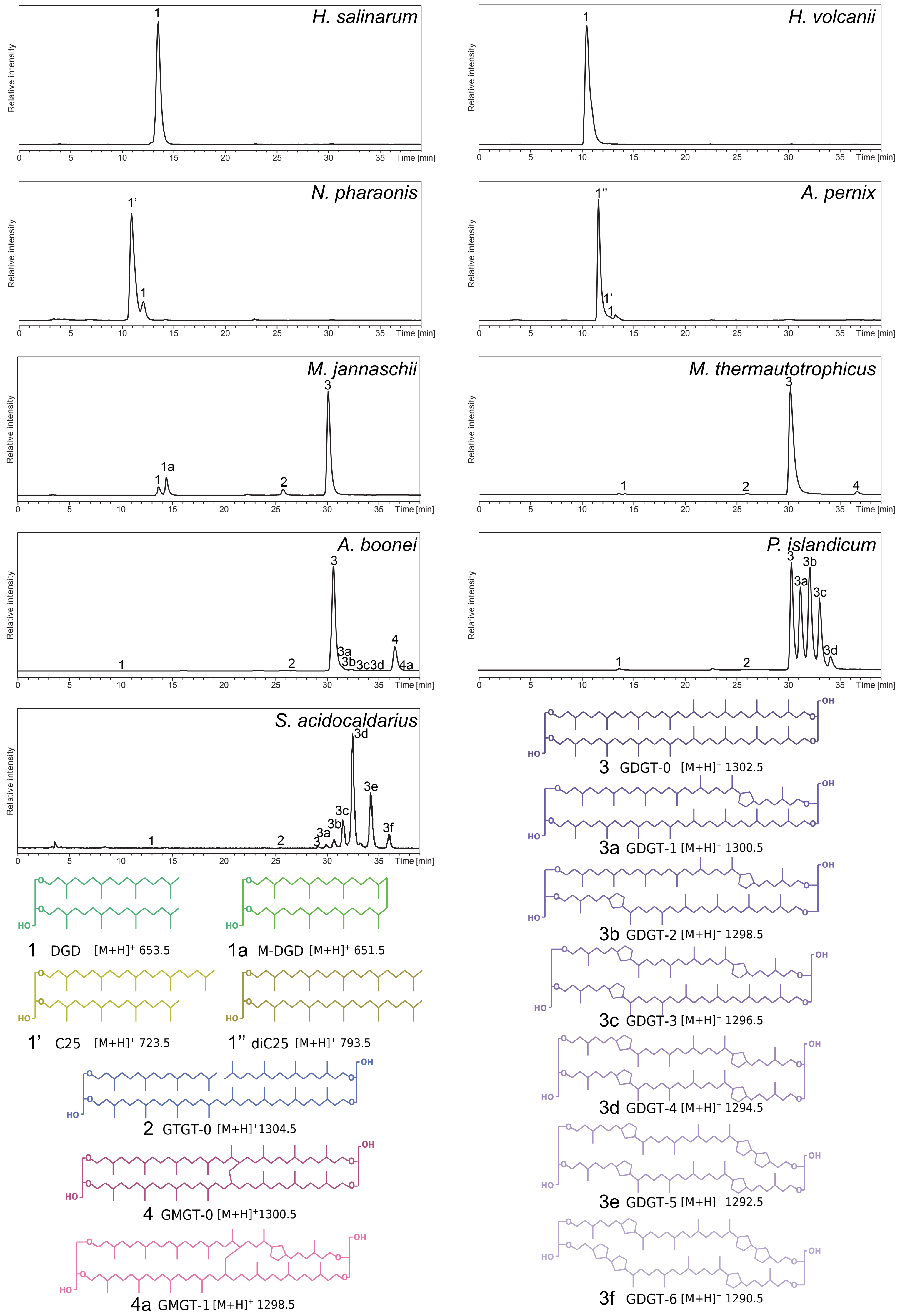

Figure S1. HPLC chromatograms (top) and lipid core structures (bottom) identified in *Halobacterium salinarum*, *Haloferax volcanii*, *Natronomonas pharaonis*, *Aeropyrum pernix*, *Methanocaldococcus jannaschii*, *Methanothermobacter thermautotrophicus*, *Aciduliprofundum boonei*, *Pyrobaculum islandicum* and *Sulfolobus acidocaldarius*. Cells were grown under their respective optimal conditions as described in the literature. (1) Diphytanyl glycerol diether (DGD, green), (1a) Macrocyclic DGD (M-DGD, light green), (1′) 2-O-phytanyl-3-O-sesterterpanyl-sn-glycerol and/or 2-O-sesterterpanyl-3-O-phytanyl-sn-glycerol diether (C25, light yellow), (1″) disesterterpanyl glycerol diether (diC25, dark yellow), (2) glycerol trialkyl glycerol tetraethers with no cyclopentane ring (GTGT-0, blue), (3 to 3f) glycerol dibiphytanyl glycerol tetraethers with 0 to 6 rings (GDGT-0 to -6, dark to light purple) and (4 and 4a) glycerol monoalkyl glycerol tetraethers with 0 (GMGT-0, dark red) and 1 ring (GMGT-1, light red). [M+H+]: mass/charge ratio.

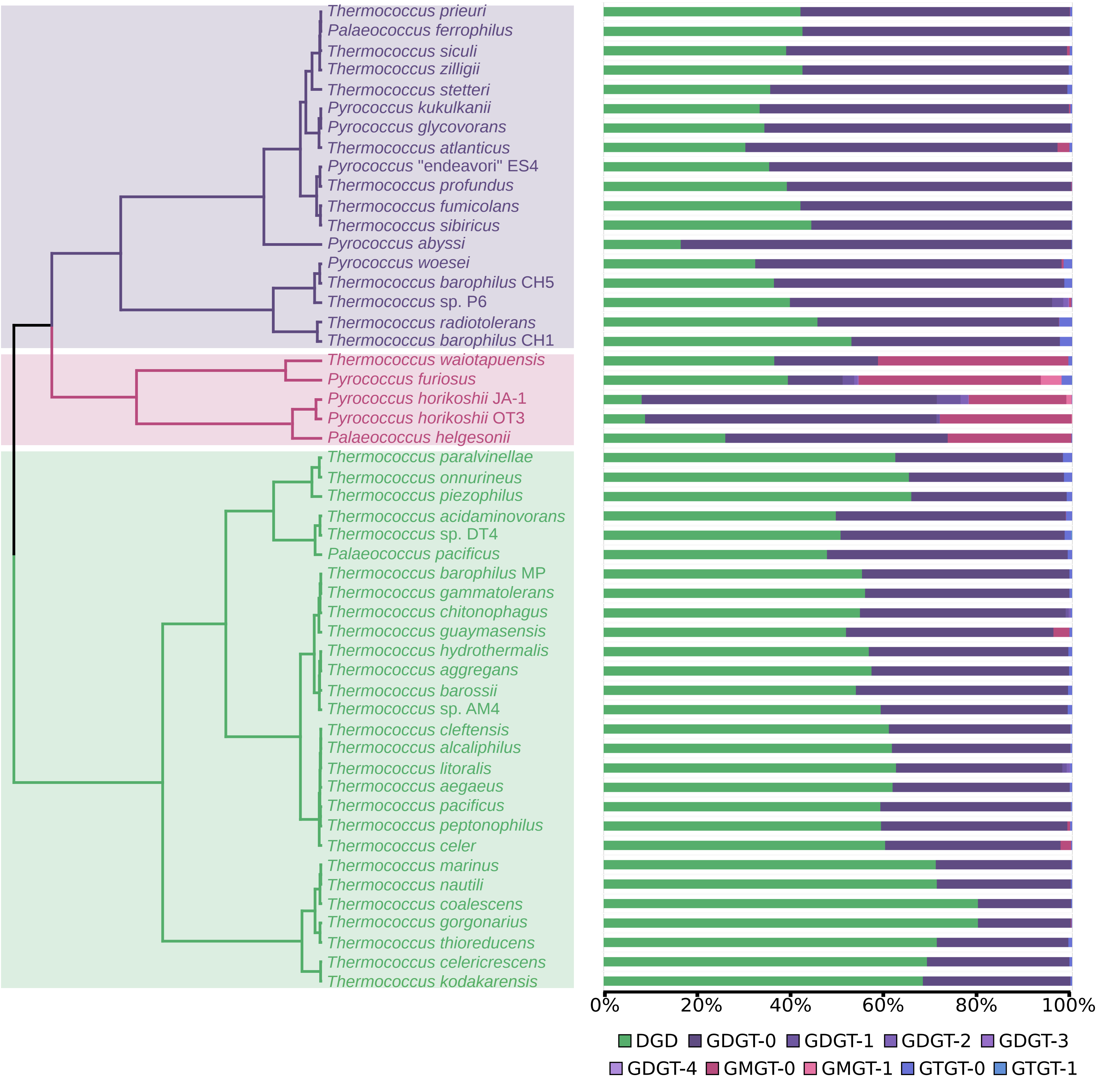

Figure S2. Thermococcales core lipid composition clustering. A hierarchical clustering on principal components was conducted with the FactoMineR (Lê et al., 2008) R package (version 1.38) using Euclidean distance and the average linkage method on the mean core lipid abundances without prior data scaling. The optimal number of clusters was automatically computed using the highest relative loss of inertia.

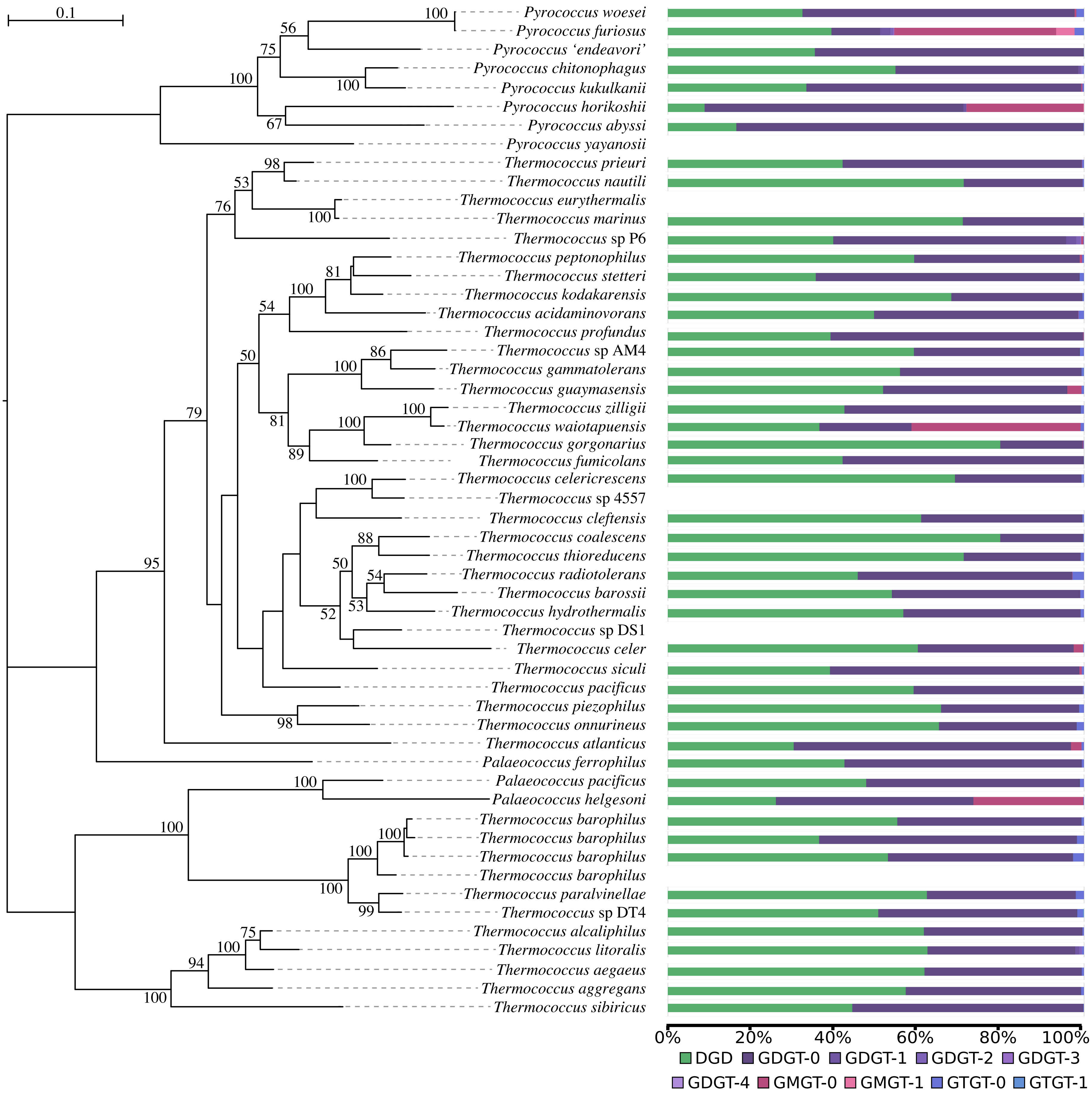

Figure S3. Thermococcales core lipid composition vs. lipid synthesis gene phylogeny. Unrooted ML phylogeny of *Thermococcales* was inferred from the aligned concatenated protein sequences of the five core genes of archaeal lipid synthesis, e.g. the CDR archaeaol synthase (TERMP_01811), the (S)-3-O-geranylgeranylglyceryl phosphate synthase (TERMP_01020), (S)-2,3-di-O-geranylgeranylglyceryl phosphate synthase (TERMP_02000), the Digeranylgeranylglyceryl phospholipid reductase (TERMP_00654) and the multifunctional geranyl diphosphate/farnesyl diphosphate/geranylgeranyl diphosphate synthase (TERMP_00147) (1,467 amino acid positions). The tree was inferred with PhyML (LG). The scale bar represents the average number of substitutions per site. Numbers at branches represent bootstrap values (1000 replicates, values > 50% are shown). Core lipid relative proportions from Table 1 are represented as bar charts using the same color code as that in Figure 1.

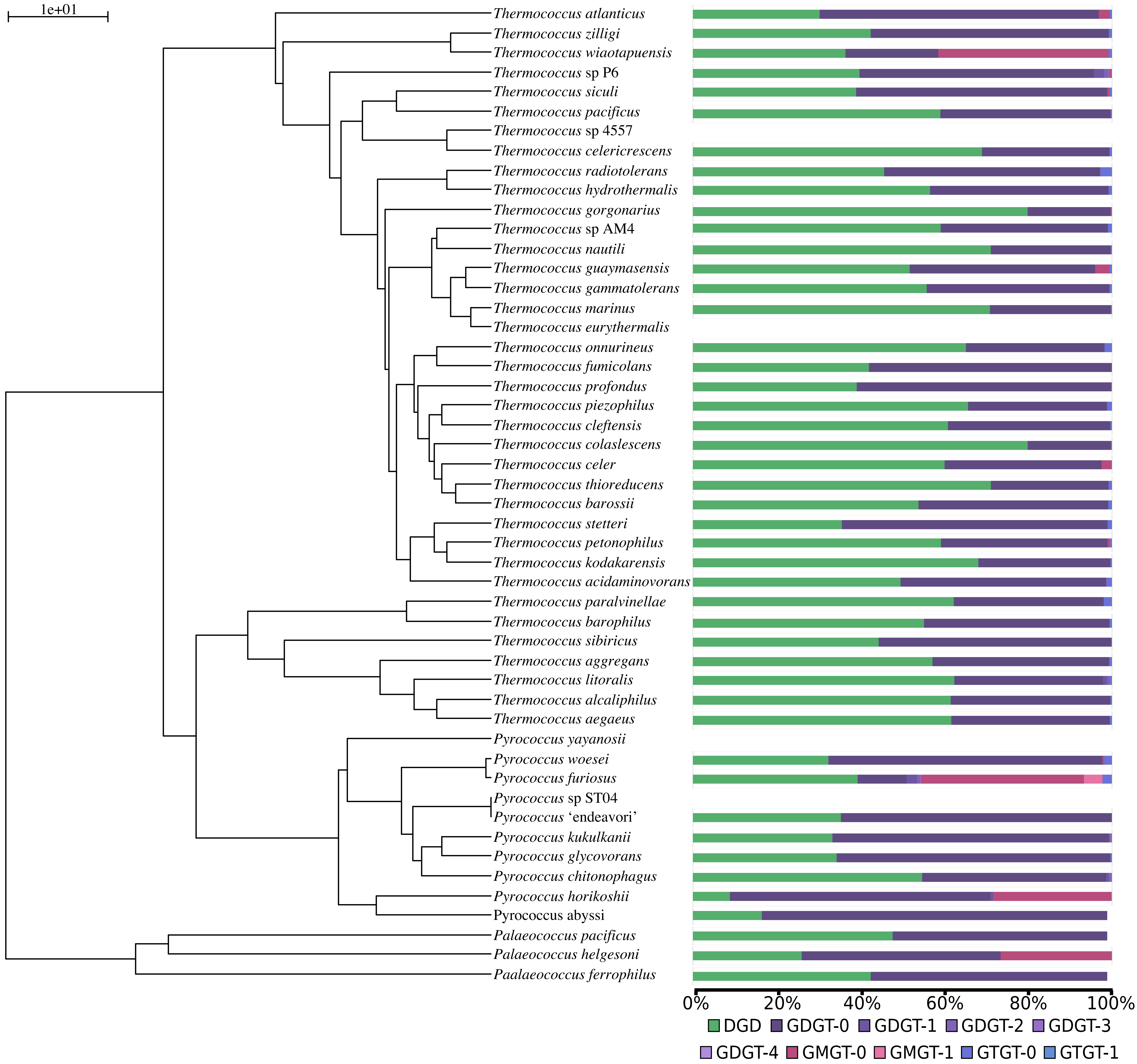

Figure S4. Thermococcales core lipid composition vs. whole genome phylogeny. Unrooted phylogeny of *Thermococcales* was inferred from whole genomes suing REALPHY. The scale bar represents the average number of substitutions per site. Core lipid relative proportions from Table 1 are represented as bar charts using the same color code as that in Figure 1.

1.2 Supplementary Tables

Table S1. Original site characteristics and cultivation conditions of the 51 *Thermococcales* strains analyzed. (XLSX format)

Table S2. Literature survey of the lipid compositions of 440 archaeal species. (XLSX format)

Table S3. Core lipid composition of the 9 additional strains. For each strain, values are the average of 3 biological replicates, except for *M. thermautotrophicus* which core lipid composition has been assessed only once (relative % ± standard deviation). (XLSX format)

2 Supplementary References

Albuquerque, L., Kowalewicz-Kulbat, M., Drzewiecka, D., Staczek, P., d'Auria, G., Rosselló-Móra, R., et al. (2016). Halorhabdus rudnickae sp. nov., a halophilic archaeon isolated from a salt mine borehole in Poland. *Syst. Appl. Microbiol.* 39, 100–105. doi:10.1016/j.syapm.2015.12.004.

Albuquerque, L., Taborda, M., La Cono, V., Yakimov, M., and Da Costa, M. S. (2012). Natrinema salaciae sp. Nov., a halophilic archaeon isolated from the deep, hypersaline anoxic Lake Medee in the Eastern Mediterranean Sea. *Syst. Appl. Microbiol.* 35, 368–373. doi:10.1016/j.syapm.2012.06.005.

Allen, M. A., Goh, F., Leuko, S., Echigo, A., Mizuki, T., Usami, R., et al. (2008). Haloferax elongans sp. nov. and Haloferax mucosum sp. nov., isolated from microbial mats from Hamelin Pool, Shark Bay, Australia. *Int. J. Syst. Evol. Microbiol.* 58, 798–802. doi:10.1099/ijs.0.65360-0.

Amoozegar, M. A., Makhdoumi-Kakhki, A., Fazeli, S. A. S., Azarbaijani, R., and Ventosa, A. (2012). Halopenitus persicus gen. nov., sp. nov., an archaeon from an Inland salt lake. *Int. J. Syst. Evol. Microbiol.* 62, 1932–1936. doi:10.1099/ijs.0.036921-0.

Amoozegar, M. A., Makhdoumi-Kakhki, A., Mehrshad, M., Fazeli, S. A. S., Sproer, C., and Ventosa, A. (2014a). Halorientalis persicus sp. nov., an extremely halophilic archaeon isolated from a salt lake and emended description of the genus Halorientalis. *Int. J. Microbiol.* 64, 940–944. doi:10.1099/ijs.0.058164-0.

Amoozegar, M. A., Makhdoumi-Kakhki, A., Mehrshad, M., Rasooli, M., Fazeli, S. A. S., Spröer, C., et al. (2015). Halovivax cerinus sp. nov., an extremely halophilic archaeon from a hypersaline lake. *Int. J. Syst. Evol. Microbiol.* 65, 65–70. doi:10.1099/ijs.0.069757-0.

Amoozegar, M. A., Makhdoumi-Kakhki, A., Mehrshad, M., Riazi, S., and Ventosa, A. (2014b). Halovivax limisalsi sp. nov., an extremely halophilic archaeon from hypersaline mud. *Int. J. Syst. Evol. Microbiol.* 64, 3422–3426. doi:10.1099/ijs.0.063297-0.

Amoozegar, M. A., Makhdoumi-Kakhki, A., Mehrshad, M., Shahzadeh Fazeli, S. A., and Ventosa, A. (2013). Halopenitus malekzadehii sp. nov., an extremely halophilic archaeon isolated from a salt lake. *Int. J. Syst. Evol. Microbiol.* 63, 3232–3236. doi:10.1099/ijs.0.050500-0.

Antunes, A., Taborda, M., Huber, R., Moissl, C., Nobre, M. F., and da Costa, M. S. (2008). Halorhabdus tiamatea sp. nov., a non-pigmented extremely halophilic archaeon from a deep-sea hypersaline anoxic basin of the Red Sea, and emended description of the genus Halorhabdus. *Int. J. Syst. Evol. Microbiol.* 58, 215–220. doi:10.1099/ijs.0.65316-0.

Asker, D., and Ohta, Y. (2002). Haloferax alexandrinus sp. nov., an extremely halophilic canthaxanthin-producing archaeon from a solar saltern in Alexandria (Egypt). *Int. J. Syst. Evol. Microbiol.* 52, 729–738. doi:10.1099/00207713-52-3-729.

Bale, N. J., Sorokin, D. Y., Hopmans, E. C., Koenen, M., Irene Rijpstra, W. C., Villanueva, L., et al. (2019). New insights into the polar lipid composition of extremely halo(alkali)philic euryarchaea from hypersaline lakes. *Front. Microbiol.* 10. doi:10.3389/fmicb.2019.00377.

Bardavid, R. E., Mana, L., and Oren, A. (2007). Haloplanus natans gen. nov., sp. nov., an extremely

halophilic, gas-vacuolate archaeon isolated from Dead Sea-Red Sea water mixtures in experimental outdoor ponds. *Int. J. Syst. Evol. Microbiol.* 57, 780–783. doi:10.1099/ijs.0.64648-0.

Bauersachs, T., Weidenbach, K., Schmitz, R. A., and Schwark, L. (2015). Distribution of glycerol ether lipids in halophilic, methanogenic and hyperthermophilic archaea. *Org. Geochem.* 83–84, 101–108. doi:10.1016/j.orggeochem.2015.03.009.

Baumann, L. M. F., Taubner, R.-S., Bauersachs, T., Steiner, M., Schleper, C., Peckmann, J., et al. (2018). Intact polar lipid and core lipid inventory of the hydrothermal vent methanogens Methanocaldococcus villosus and Methanothermococcus okinawensis. *Org. Geochem.* 126, 33–42. doi:10.1016/j.orggeochem.2018.10.006.

Becker, K. W., Elling, F. J., Yoshinaga, M. Y., Söllinger, A., Urich, T., and Hinrichs, K. U. (2016). Unusual butane- and pentanetriol-based tetraether lipids in Methanomassiliicoccus Luminyensis, a representative of the seventh order of methanogens. *Appl. Environ. Microbiol.* 82, 4505–4516. doi:10.1128/AEM.00772-16.

Blöchl, E., Stetter, R., Burggraf, S., Hafenbradl, D., Jannasch, H. W., and Stetter, K. O. (1997). Pyrolobus fumarii, gen. and sp. nov., represents a novel group of archaea, extending the upper temperature limit for life to 113 degrees C. *Extremophiles* 1, 14–21.

Blotevogel, K.-H., Gahl-Janssen, R., Jannsen, S., Fischer, U., Pilz, F., Auling, G., et al. (1991). Isolation and characterization of a novel mesophilic, fresh-water methanogen from river sediment Methanoculleus oldenburgensis sp. nov. *Arch. Microbiol.* 157, 54–59. doi:10.1007/BF00245335.

Boulahrouf, A., Ramos-Cormenzana, A., Kharroub, K., Aguilera, M., Monteoliva-Sanchez, M., Lizama, C., et al. (2008). Halomicrobium katesii sp. nov., an extremely halophilic archaeon. *Int. J. Syst. Evol. Microbiol.* 58, 2354–2358. doi:10.1099/ijs.0.65662-0.

Boyd, E. S., Jackson, R. A., Encarnacion, G., Zahn, J. A., Beard, T., Leavitt, W. D., et al. (2007). Isolation, characterization, and ecology of sulfur-respiring crenarchaea inhabiting acid-sulfate-chloride-containing geothermal springs in Yellowstone National Park. *Appl. Environ. Microbiol.* 73, 6669–6677. doi:10.1128/aem.01321-07.

Bräuer, S. L., Cadillo-Quiroz, H., Ward, R. J., Yavitt, J. B., and Zinder, S. H. (2011). Methanoregula boonei gen. nov., sp. nov., an acidiphilic methanogen isolated from an acidic peat bog. *Int. J. Syst. Evol. Microbiol.* 61, 45–52. doi:10.1099/ijs.0.021782-0.

Burggraf, S., Jannasch, H. W., Nicolaus, B., and Stetter, K. O. (1990). Archaeoglobus profundus sp. nov., Represents a New Species within the Sulfate-reducing Archaebacteria. *Syst. Appl. Microbiol.* 13, 24–28. doi:10.1016/S0723-2020(11)80176-1.

Burns, D. G., Janssen, P. H., Itoh, T., Kamekura, M., Echigo, A., and Dyall-Smith, M. L. (2010a). Halonotius pteroides gen. nov., sp. nov., an extremely halophilic archaeon recovered from a saltern crystallizer. *Int. J. Syst. Evol. Microbiol.* 60, 1196–1199. doi:10.1099/ijs.0.010017-0.

Burns, D. G., Janssen, P. H., Itoh, T., Kamekura, M., Li, Z., Jensen, G., et al. (2007). Haloquadratum walsbyi gen. nov., sp. nov., the square haloarchaeon of Walsby, isolated from saltern

crystallizers in Australia and Spain. *Int. J. Syst. Evol. Microbiol.* 57, 387–392. doi:10.1099/ijs.0.64690-0.

Burns, D. G., Janssen, P. H., Itoh, T., Minegishi, H., Usami, R., Kamekura, M., et al. (2010b). Natronomonas moolapensis sp. nov., non-alkaliphilic isolates recovered from a solar saltern crystallizer pond, and emended description of the genus Natronomonas. *Int. J. Syst. Evol. Microbiol.* 60, 1173–1176. doi:10.1099/ijs.0.010132-0.

Callac, N., Oger, P., Lesongeur, F., Rattray, J. E., Vannier, P., Michoud, G., et al. (2016). Pyrococcus kukulkanii sp. nov., a hyperthermophilic, piezophilic archaeon isolated from a deep-sea hydrothermal vent. *Int. J. Syst. Evol. Microbiol.* 66, 3142–3149. doi:10.1099/ijsem.0.001160.

Cario, A., Grossi, V., Schaeffer, P., and Oger, P. M. (2015). Membrane homeoviscous adaptation in the piezo-hyperthermophilic archaeon Thermococcus barophilus. *Front. Microbiol.* 6, 1–12. doi:10.3389/fmicb.2015.01152.

Castillo, A. M., Gutiérrez, M. C., Kamekura, M., Ma, Y., Cowan, D. A., Jones, B. E., et al. (2006a). Halovivax asiaticus gen. nov., sp. nov., a novel extremely halophilic archaeon isolated from Inner Mongolia, China. *Int. J. Syst. Evol. Microbiol.* 56, 765–770. doi:10.1099/ijs.0.63954-0.

Castillo, A. M., Gutierrez, M. C., Kamekura, M., Xue, Y., Ma, Y., Cowan, D. A., et al. (2006b). Halorubrum orientale sp. nov., a halophilic archaeon isolated from Lake Ejinor, Inner Mongolia, China. *Int. J. Syst. Evol. Microbiol.* 56, 2559–2563. doi:10.1099/ijs.0.64420-0.

Castillo, A. M., Gutierrez, M. C., Kamekura, M., Xue, Y., Ma, Y., Cowan, D. A., et al. (2007a). Halovivax ruber sp. nov., an extremely halophilic archaeon isolated from Lake Xilinhot, Inner Mongolia, China. *Int. J. Syst. Evol. Microbiol.* 57, 1024–1027. doi:10.1099/ijs.0.64899-0.

Castillo, A. M., Gutiérrez, M. C., Kamekura, M., Xue, Y., Ma, Y., Cowan, D. A., et al. (2006c). Halostagnicola larsenii gen. nov., sp. nov., an extremely halophilic archaeon from a saline lake in Inner Mongolia, China. *Int. J. Syst. Evol. Microbiol.* 56, 1519–1524. doi:10.1099/ijs.0.64286-0.

Castillo, A. M., Gutiérrez, M. C., Kamekura, M., Xue, Y., Ma, Y., Cowan, D. A., et al. (2006d). Natrinema ejinorense sp. nov., isolated from a saline lake in Inner Mongolia, China. *Int. J. Syst. Evol. Microbiol.* 56, 2683–2687. doi:10.1099/ijs.0.64421-0.

Castillo, A. M., Gutiérrez, M. C., Kamekura, M., Xue, Y., Ma, Y., Cowan, D. A., et al. (2007b). Halorubrum ejinorense sp. nov., isolated from Lake Ejinor, Inner Mongolia, China. *Int. J. Syst. Evol. Microbiol.* 57, 2538–2542. doi:10.1099/ijs.0.65241-0.

Cha, I. T., Yim, K. J., Song, H. S., Lee, H. W., Hyun, D. W., Kim, K. N., et al. (2014a). Halobellus rufus sp. nov., an extremely halophilic archaeon isolated from non-purified solar salt. *Antonie van Leeuwenhoek, Int. J. Gen. Mol. Microbiol.* 105, 925–932. doi:10.1007/s10482-014-0147-y.

Cha, I. T., Yim, K. J., Song, H. S., Lee, H. W., Hyun, D. W., Kim, K. N., et al. (2014b). Halolamina rubra sp. nov., a haloarchaeon isolated from non-purified solar salt. *Antonie van Leeuwenhoek, Int. J. Gen. Mol. Microbiol.* 105, 907–914. doi:10.1007/s10482-014-0145-0.

Charlou, J. L., Donval, J. P., Fouquet, Y., Jean-baptiste, P., and Holm, N. (2002). Geochemistry of

high H\textsubscript{2} and CH\textsubscript{4} vent fluids issuing from ultramafic rocks at the Rainbow hydrothermal field (36°14' N, MAR). *Chem. Geol.* 191, 345–359. doi:10.1016/S0009-2541(02)00134-1.

Chen, S., Liu, H. C., Zhao, D., Yang, J., Zhou, J., and Xiang, H. (2015). Halorubrum yunnanense sp. Nov., isolated from a subterranean salt mine. *Int. J. Syst. Evol. Microbiol.* 65, 4526–4532. doi:10.1099/ijsem.0.000605.

Chen, S., Liu, H. C., Zhou, J., and Xiang, H. (2016a). Haloparvum sedimenti gen. nov., sp. nov., a member of the family Haloferacaceae. *Int. J. Syst. Evol. Microbiol.* 66, 2327–2334. doi:10.1099/ijsem.0.001033.

Chen, S., Liu, H. C., Zhou, J., and Xiang, H. (2016b). Halorubrum pallidum sp. nov., an extremely halophilic archaeon isolated from a subterranean rock salt. *Int. J. Syst. Evol. Microbiol.* 66, 2980–2986. doi:10.1099/ijsem.0.001129.

Chen, S., Sun, S., Xu, Y., and Liu, H.-C. (2018). Halococcus salsus sp. nov., a novel halophilic archaeon isolated from rock salt. *Int. J. Syst. Evol. Microbiol.* 68, 3754–3759. doi:10.1099/ijsem.0.003051.

Chen, S., Xu, Y., and Ke, L. X. (2017a). Halorubrum trueperi sp. nov., a halophilic archaeon isolated from a salt mine. *Int. J. Syst. Evol. Microbiol.* 67, 1564–1570. doi:10.1099/ijsem.0.001762.

Chen, S., Xu, Y., Liu, H. C., Yang, A. N., and Ke, L. X. (2017b). Halobaculum roseum sp. nov., isolated from underground salt deposits. *Int. J. Syst. Evol. Microbiol.* 67, 818–823. doi:10.1099/ijsem.0.001663.

Cho, E.-S., Cha, I.-T., Roh, S. W., Nam, Y.-D., and Seo, M.-J. (2018). Haloplanus rallus sp. nov., a halophilic archaeon isolated from crude solar salt. *Int. J. Syst. Evol. Microbiol.* 68, 3226–3231. doi:10.1099/ijsem.0.002970.

Choquet, C. G., Patel, G. B., Beveridge, T. J., and Sprott, G. D. (1992). Formation of unilamellar liposomes from total polar lipid extracts of methanogens. *Appl. Environ. Microbiol.* 58, 2894–2900.

Choquet, C. G., Patel, G. B., Sprott, G. D., and Beveridge, T. J. (1994). Stability of pressure-extruded liposomes made from archaeobacterial ether lipids. *Appl. Microbiol. Biotechnol.* 42, 375–384. doi:10.1007/BF00902745.

Corral, P., Corcelli, A., and Ventosa, A. (2015a). Halostagnicola bangensis sp. nov., an alkaliphilic haloarchaeon from a soda lake. *Int. J. Syst. Evol. Microbiol.* 65, 754–759. doi:10.1099/ijs.0.000006.

Corral, P., de la Haba, R. R., Sánchez-Porro, C., Amoozegar, M. A., Papke, R. T., and Ventosa, A. (2016). Halorubrum halodurans sp. Nov., an extremely halophilic archaeon isolated from a hypersaline lake. *Int. J. Syst. Evol. Microbiol.* 66, 435–444. doi:10.1099/ijsem.0.000738.

Corral, P., De La Haba, R. R., Sánchez-Porro, C., Amoozegar, M. A., Thane Papke, R., and Ventosa, A. (2015b). Halorubrum persicum sp. nov., an extremely halophilic archaeon isolated from sediment of a hypersaline lake. *Int. J. Syst. Evol. Microbiol.* 65, 1770–1778.

doi:10.1099/ijs.0.000175.

Corral, P., Gutiérrez, M. C., Castillo, A. M., Domínguez, M., Lopalco, P., Corcelli, A., et al. (2013). Natronococcus roseus sp. nov., a haloalkaliphilic archaeon from a hypersaline lake. *Int. J. Syst. Evol. Microbiol.* 63, 104–108. doi:10.1099/ijs.0.036558-0.

Cui, H.-L. (2006). Natronorubrum aibiense sp. nov., an extremely halophilic archaeon isolated from Aibi salt lake in Xin-Jiang, China, and emended description of the genus Natronorubrum. *Int. J. Syst. Evol. Microbiol.* 56, 1515–1517. doi:10.1099/ijs.0.64222-0.

Cui, H. L., Gao, X., Li, X. Y., Xu, E. W., Zhou, Y. G., Liu, H. C., et al. (2010a). Haloplanus vescus sp. nov., an extremely halophilic archaeon from a marine solar saltern, and emended description of the genus Haloplanus. *Int. J. Syst. Evol. Microbiol.* 60, 1824–1827. doi:10.1099/ijs.0.018564-0.

Cui, H. L., Gao, X., Sun, F. F., Dong, Y., Xu, X. W., Zhou, Y. G., et al. (2010b). Halogranum rubrum gen. nov., sp. nov., a halophilic archaeon isolated from a marine solar saltern. *Int. J. Syst. Evol. Microbiol.* 60, 1366–1371. doi:10.1099/ijs.0.014928-0.

Cui, H. L., Gao, X., Yang, X., and Xu, X. W. (2010c). Halorussus rarus gen. nov., sp. nov., a new member of the family Halobacteriaceae isolated from a marine solar saltern. *Extremophiles* 14, 493–499. doi:10.1007/s00792-010-0329-0.

Cui, H. L., Gao, X., Yang, X., and Xu, X. W. (2011a). Halolamina pelagica gen. nov., sp. nov., a new member of the family Halobacteriaceae. *Int. J. Syst. Evol. Microbiol.* 61, 1617–1621. doi:10.1099/ijs.0.026799-0.

Cui, H. L., Gao, X., Yang, X., and Xu, X. W. (2011b). Haloplanus aerogenes sp. nov., an extremely halophilic archaeon from a marine solar saltern. *Int. J. Syst. Evol. Microbiol.* 61, 965–968. doi:10.1099/ijs.0.025023-0.

Cui, H. L., Li, X. Y., Gao, X., Xu, X. W., Zhou, Y. G., Liu, H. C., et al. (2010d). Halopelagius inordinatus gen. nov., sp. nov., a new member of the family Halobacteriaceae isolated from a marine solar saltern. *Int. J. Syst. Evol. Microbiol.* 60, 2089–2093. doi:10.1099/ijs.0.018598-0.

Cui, H. L., Lin, Z. Y., Dong, Y., Zhou, P. J., and Liu, S. J. (2007a). Halorubrum litoreum sp. nov., an extremely halophilic archaeon from a solar saltern. *Int. J. Syst. Evol. Microbiol.* 57, 2204–2206. doi:10.1099/ijs.0.65268-0.

Cui, H. L., Lü, Z. Z., Li, Y., and Zhou, Y. (2017). Salinirussus salinus gen. Nov., sp. nov., isolated from a marine solar saltern. *Int. J. Syst. Evol. Microbiol.* 67, 3622–3626. doi:10.1099/ijsem.0.002182.

Cui, H. L., Mou, Y. Z., Yang, X., Zhou, Y. G., Liu, H. C., and Zhou, P. J. (2012a). Halorubellus salinus gen. nov., sp. nov. and Halorubellus litoreus sp. nov., novel halophilic archaea isolated from a marine solar saltern. *Syst. Appl. Microbiol.* 35, 30–34. doi:10.1016/j.syapm.2011.08.001.

Cui, H. L., and Qiu, X. X. (2014). Salinarubrum litoreum gen. Nov., sp. Nov.: A new member of the family Halobacteriaceae isolated from Chinese marine solar salterns. *Antonie van Leeuwenhoek, Int. J. Gen. Mol. Microbiol.* 105, 135–141. doi:10.1007/s10482-013-0061-8.

Cui, H. L., Sun, F. F., Gao, X., Dong, Y., Xu, E. W., Zhou, Y. G., et al. (2010e). Haladaptatus litoreus sp. nov., an extremely halophilic archaeon from a marine solar saltern, and emended description of the genus Haladaptatus. *Int. J. Syst. Evol. Microbiol.* 60, 1085–1089. doi:10.1099/ijs.0.015933-0.

Cui, H. L., Tohty, D., Liu, H. C., Liu, S. J., Oren, A., and Zhou, P. J. (2007b). Natronorubrum sulfidifaciens sp. nov., an extremely haloalkaliphilic archaeon isolated from Aiding salt lake in Xin-Jiang, China. *Int. J. Syst. Evol. Microbiol.* 57, 738–740. doi:10.1099/ijs.0.64651-0.

Cui, H. L., Tohty, D., Zhou, P. J., and Liu, S. J. (2006a). Halorubrum lipolyticum sp. nov. and Halorubrum aidingense sp. nov., isolated from two salt lakes in Xin-Jiang, China. *Int. J. Syst. Evol. Microbiol.* 56, 1631–1634. doi:10.1099/ijs.0.64305-0.

Cui, H. L., Tohty, D., Zhou, P. J., and Liu, S. J. (2006b). Haloterrigena longa sp. nov. and Haloterrigena limicola sp. nov., extremely halophilic archaea isolated from a salt lake. *Int. J. Syst. Evol. Microbiol.* 56, 1837–1840. doi:10.1099/ijs.0.64372-0.

Cui, H. L., Yang, X., Gao, X., and Xu, X. W. (2011c). Halobellus clavatus gen. nov., sp. nov. and Halorientalis regularis gen. nov., sp. nov., two new members of the family Halobacteriaceae. *Int. J. Syst. Evol. Microbiol.* 61, 2682–2689. doi:10.1099/ijs.0.025841-0.

Cui, H. L., Yang, X., Gao, X., and Xu, X. W. (2011d). Halogranum gelatinilyticum sp. nov. and Halogranum amylolyticum sp. nov., isolated from a marine solar saltern, and emended description of the genus Halogranum. *Int. J. Syst. Evol. Microbiol.* 61, 911–915. doi:10.1099/ijs.0.024976-0.

Cui, H. L., Yang, X., and Mou, Y. Z. (2011e). Salinarchaeum laminariae gen. nov., sp. nov.: A new member of the family Halobacteriaceae isolated from salted brown alga Laminaria. *Extremophiles* 15, 625–631. doi:10.1007/s00792-011-0393-0.

Cui, H. L., Yang, X., Zhou, Y. G., Liu, H. C., Zhou, P. J., and Dyall-Smith, M. L. (2012b). Halobellus limi sp. nov. and Halobellus salinus sp. nov., isolated from two marine solar salterns. *Int. J. Syst. Evol. Microbiol.* 62, 1307–1313. doi:10.1099/ijs.0.032169-0.

Cui, H. L., and Zhang, W. J. (2014). Salinigranum rubrum gen. nov., sp. nov., a member of the family Halobacteriaceae isolated from a marine solar saltern. *Int. J. Syst. Evol. Microbiol.* 64, 2029–2033. doi:10.1099/ijs.0.061606-0.

De Rosa, M., Gambacorta, A., and Gliozzi, A. (1986). Structure, biosynthesis, and physicochemical properties of archaebacterial lipids. *Microbiol. Rev.* 50, 70–80. Available at: http://www.ncbi.nlm.nih.gov/pubmed/3083222.

De Rosa, M., Gambacorta, A., Nicolaus, B., Chappe, B., and Albrecht, P. (1983). Isoprenoid ethers; backbone of complex lipids of the archaeabacterium Sulfolobus acidocaldarius. 753, 249–256. Available at: http://ac.els-cdn.com/0005276083900140/1-s2.0-0005276083900140-main.pdf?_tid=2d88fcf2-bf2f-11e2-bd1b-00000aacb360&acdnat=1368822192_d3422d8055c513cb2a1a67f7b94d88ed%0Apapers2://publication/uuid/D13F59CF-3D25-4C39-9074-884D2FF5F7ED%0Apapers2://publication/.

De Rosa, M., Gambacorta, A., Trincone, A., Basso, A., Zillig, W., and Holz, I. (1987). Lipids of Thermococcus celer, a sulfur-reducing archaebacterium: Structure and biosynthesis. *Syst. Appl. Microbiol.* 9, 1–5. doi:10.1016/S0723-2020(87)80046-2.

de Souza, L. M., Müller-Santos, M., Iacomini, M., Gorin, P. A. J., and Sassaki, G. L. (2009). Positive and negative tandem mass spectrometric fingerprints of lipids from the halophilic Archaea Haloarcula marismortui. *J. Lipid Res.* 50, 1363–1373. doi:10.1194/jlr.M800478-JLR200.

Denner, E. B. M., McGenity, T. J., Busse, H.-J., Grant, W. D., Wanner, G., and Stan-Lotter, H. (1994). Halococcus salifodinae sp. nov., an Archaeal Isolate from an Austrian Salt Mine. *Int. J. Syst. Bacteriol.* 44, 774–780. doi:10.1099/00207713-44-4-774.

Dianou, D., Miyaki, T., Asakawa, S., Morii, H., Nagaoka, K., Oyaizu, H., et al. (2001). Methanoculleus chikugoensis sp. nov., a novel methanogenic archaeon isolated from paddy field soil in Japan, and DNA-DNA hybridization among Methanoculleus species. *Int. J. Syst. Evol. Microbiol.* 51, 1663–1669. doi:10.1099/00207713-51-5-1663.

Ding, J. Y., Chen, S. C., Lai, M. C., and Liao, T. L. (2017). Haloterrigena mahii sp. nov., an extremely halophilic archaeon from a solar saltern. *Int. J. Syst. Evol. Microbiol.* 67, 1333–1338. doi:10.1099/ijsem.0.001811.

Dirmeier, R., Keller, M., Hafenbradl, D., Braun, F.-J., Rachel, R., Burggraf, S., et al. (1998). Thermococcus acidaminovorans sp. nov., a new hyperthermophilic alkalophilic archaeon growing on amino acids. *Extremophiles* 2, 109–114. doi:10.1007/s007920050049.

Echigo, A., Minegishi, H., Shimane, Y., Kamekura, M., Itoh, T., and Usami, R. (2013). Halomicroarcula pellucida gen. nov., sp. nov., a non-pigmented, transparent-colony-forming, halophilic archaeon isolated from solar salt. *Int. J. Syst. Evol. Microbiol.* 63, 3556–3562. doi:10.1099/ijs.0.049965-0.

Elling, F. J., Könneke, M., Nicol, G. W., Stieglmeier, M., Bayer, B., Spieck, E., et al. (2017). Chemotaxonomic characterisation of the thaumarchaeal lipidome. *Environ. Microbiol.* 19, 2681–2700. doi:10.1111/1462-2920.13759.

Elshahed, M. S., Savage, K. N., Oren, A., Gutierrez, M. C., Ventosa, A., and Krumholz, L. R. (2004). Haloferax sulfurifontis sp. nov., a halophilic archaeon isolated from a sulfide- and sulfur-rich spring. *Int. J. Syst. Evol. Microbiol.* 54, 2275–2279. doi:10.1099/ijs.0.63211-0.

Enache, M., Itoh, T., Kamekura, M., Teodosiu, G., and Dumitru, L. (2007). Haloferax prahovense sp. nov., an extremely halophilic archaeon isolated from a Romanian salt lake. *Int. J. Syst. Evol. Microbiol.* 57, 393–397. doi:10.1099/ijs.0.64674-0.

Erauso, G., Reysenbach, A.-L., Godfroy, A., Meunier, J.-R., Crump, B., Partensky, F., et al. (1993). Pyrococcus abyssi sp. nov., a new hyperthermophilic archaeon isolated from a deep-sea hydrothermal vent. *Arch. Microbiol.* 160, 205–209. doi:10.1007/BF00252219.

Erdmann, S., Chen, B., Huang, X., Deng, L., Liu, C., Shah, S. A., et al. (2014). A novel single-tailed fusiform Sulfolobus virus STSV2 infecting model Sulfolobus species. *Extremophiles* 18, 51–60. doi:10.1007/s00792-013-0591-z.

Fan, H., Xue, Y., Ma, Y., Ventosa, A., and Grant, W. D. (2004). Halorubrum tibetense sp. nov., a novel haloalkaliphilic archaeon from Lake Zabuye in Tibet, China. *Int. J. Syst. Evol. Microbiol.* 54, 1213–1216. doi:10.1099/ijs.0.03032-0.

Feng, J., Zhou, P. J., and Liu, S. J. (2004). Halorubrum xinjiangense sp. nov., a novel halophile isolated from saline lakes in China. *Int. J. Syst. Evol. Microbiol.* 54, 1789–1791. doi:10.1099/ijs.0.63209-0.

Feng, J., Zhou, P., Zhou, Y. G., Liu, S. J., and Warren-Rhodes, K. (2005). Halorubrum alkaliphilum sp. nov., a novel haloalkaliphile isolated from a soda lake in Xinjiang, China. *Int. J. Syst. Evol. Microbiol.* 55, 149–152. doi:10.1099/ijs.0.63320-0.

Ferrante, G., Brisson, J. R., Patel, G. B., Ekiel, I., and Sprott, G. D. (1989). Structures of minor ether lipids isolated from the aceticlastic methanogen, *Methanothrix concilii* GP6. *J. Lipid Res.* 30, 1601–1609. Available at: http://www.ncbi.nlm.nih.gov/pubmed/2614262.

Ferrante, G., Richards, J. C., and Sprott, G. D. (1990). Structures of polar lipids from the thermophilic, deep-sea archaeohacterium Methanococcus jannaschii. *Biochem. Cell Biol.* 68, 274–283. doi:10.1139/o90-038.

Feyhl-Buska, J., Chen, Y., Jia, C., Wang, J. X., Zhang, C. L., and Boyd, E. S. (2016). Influence of growth phase, pH, and temperature on the abundance and composition of tetraether lipids in the thermoacidophile Picrophilus torridus. *Front. Microbiol.* 7. doi:10.3389/fmicb.2016.01323.

Fiala, G., Stetter, K. O., Jannasch, H. W., Langworthy, T. A., and Madon, J. (1986). Staphylothermus marinus sp. nov. Represents a Novel Genus of Extremely Thermophilic Submarine Heterotrophic Archaebacteria Growing up to 98 °C. *Syst. Appl. Microbiol.* 8, 106–113. doi:10.1016/S0723-2020(86)80157-6.

Fitzsimons, M. F., Dando, P. R., Hughes, J. A., Thiermann, F., and Akoumianaki, I. (2003). Submarine hydrothermal brine seeps off Milos, Greece: Observations and geochemistry. 57, 1–16.

Ganzert, L., Schirmack, J., Alawi, M., Mangelsdorf, K., Sand, W., Hillebrand-Voiculescu, A., et al. (2014). Methanosarcina spelaei sp. nov., a methanogenic archaeon isolated from a floating biofilm of a subsurface sulphurous lake. *Int. J. Syst. Evol. Microbiol.* 64, 3478–3484. doi:10.1099/ijs.0.064956-0.

Gibson, J. A. E., Miller, M. R., Davies, N. W., Neill, G. P., Nichols, D. S., and Volkman, J. K. (2005). Unsaturated diether lipids in the psychrotrophic archaeon Halorubrum lacusprofundi. *Syst. Appl. Microbiol.* 28, 19–26. doi:10.1016/j.syapm.2004.09.004.

Gibtan, A., Song, H. S., Kim, J. Y., Kim, Y. B., Park, N., Park, K., et al. (2018). Halorubrum aethiopicum sp. nov., an extremely halophilic archaeon isolated from commercial rock salt. *Int. J. Syst. Evol. Microbiol.* 68, 416–422. doi:10.1099/ijsem.0.002525.

Godfroy, A., Lesongeur, F., Raguenes, G., Querellou, J., Antoine, E., Meunier, J.-R., et al. (1997). Thermococcus hydrothermalis sp. nov., a New Hyperthermophilic Archaeon Isolated from a Deep-Sea Hydrothermal Vent. *Int. J. Syst. Bacteriol.* 47, 622–626. doi:10.1099/00207713-47-3-622.

Godfroy, A., Meunier, J.-R., Guezennec, J., Lesongeur, F., Raguenes, G., Rimbault, A., et al. (1996). Thermococcus fumicolans sp. nov., a New Hyperthermophilic Archaeon Isolated from a Deep-Sea Hydrothermal Vent in the North Fiji Basin. *Int. J. Syst. Bacteriol.* 46, 1113–1119. doi:10.1099/00207713-46-4-1113.

Goh, F., Leuko, S., Allen, M. A., Bowman, J. P., Kamekura, M., Neilan, B. A., et al. (2006). Halococcus hamelinensis sp. nov., a novel halophilic archaeon isolated from stromatolites in Shark Bay, Australia. *Int. J. Syst. Evol. Microbiol.* 56, 1323–1329. doi:10.1099/ijs.0.64180-0.

Golyshina, O. V., Lünsdorf, H., Kublanov, I. V., Goldenstein, N. I., Hinrichs, K. U., and Golyshin, P. N. (2016). The novel extremely acidophilic, cell-wall-deficient archaeon Cuniculiplasma divulgatum gen. Nov., sp. nov. represents a new family, Cuniculiplasmataceae fam. nov., of the order Thermoplasmatales. *Int. J. Syst. Evol. Microbiol.* 66, 332–340. doi:10.1099/ijsem.0.000725.

Golyshina, O. V., Pivovarova, T. A., Karavaiko, G. I., Kondrat'eva, T. F., Moore, E. R. B., Abraham, W. R., et al. (2000). Ferroplasma acidiphilum gen. nov., sp. nov., an acidophilic, autotrophic, ferrous-iron-oxidizing, cell-wall-lacking, mesophilic member of the Ferroplasmaceae fam. nov., comprising a distinct lineage of the Archaea. *Int. J. Syst. Evol. Microbiol.* 50, 997–1006. doi:10.1099/00207713-50-3-997.

Golyshina, O. V., Yakimov, M. M., Lünsdorf, H., Ferrer, M., Nimtz, M., Timmis, K. N., et al. (2009). Acidiplasma aeolicum gen. nov., sp. nov., a euryarchaeon of the family Ferroplasmaceae isolated from a hydrothermal pool, and transfer of Ferroplasma cupricumulans to Acidiplasma cupricumulans comb. nov. *Int. J. Syst. Evol. Microbiol.* 59, 2815–2823. doi:10.1099/ijs.0.009639-0.

González, J. M., Masuchi, Y., Robb, F. T., Ammerman, J. W., Maeder, D. L., Yanagibayashi, M., et al. (1998). Pyrococcus horikoshii sp. nov., a hyperthermophilic archaeon isolated from a hydrothermal vent at the Okinawa Trough. *Extremophiles* 2, 123–130. doi:10.1007/s007920050051.

Grant, W. D., Pinch, G., Harris, J. E., De Rosa, M., and Gambacorta, A. (1985). Polar Lipids in Methanogen Taxonomy. *Microbiology* 131, 3277–3286. doi:10.1099/00221287-131-12-3277.

Grimaud, D., Ishibashi, J. I., Lagabrielle, Y., Auzende, J. M., and Urabe, T. (1991). Chemistry of hydrothermal fluids from the 17°S active site on the North Fiji Basin Ridge (SW Pacific). *Chem. Geol.* 93, 209–218. doi:10.1016/0009-2541(91)90114-7.

Grote, R., Li, L., Tamaoka, J., Kato, C., Horikoshi, K., and Antranikian, G. (1999). Thermococcus siculi sp. nov., a novel hyperthermophilic archaeon isolated from a deep-sea hydrothermal vent at the Mid-Okinawa Trough. *Extremophiles* 3, 55–62. doi:10.1007/s007920050099.

Gruber, C., Legat, A., Pfaffenhuemer, M., Radax, C., Weidler, G., Busse, H. J., et al. (2004). Halobacterium noricense sp. nov., an archaeal isolate from a bore core of an alpine Permian salt deposit, classification of Halobacterium sp. NRC-1 as a strain of H. salinarum and emended description of H. salinarum. *Extremophiles* 8, 431–439. doi:10.1007/s00792-004-0403-6.

Gutiérrez, M. C., Castillo, A. M., Corral, P., Kamekura, M., and Ventosa, A. (2011). Halorubrum aquaticum sp. nov., an archaeon isolated from hypersaline lakes. *Int. J. Syst. Evol. Microbiol.* 61,

1144–1148. doi:10.1099/ijs.0.025015-0.

Gutiérrez, M. C., Castillo, A. M., Corral, P., Minegishi, H., and Ventosa, A. (2010). Natronorubrum sediminis sp. nov., an archaeon isolated from a saline lake. *Int. J. Syst. Evol. Microbiol.* 60, 1802–1806. doi:10.1099/ijs.0.015602-0.

Gutiérrez, M. C., Castillo, A. M., Kamekura, M., and Ventosa, A. (2008). Haloterrigena salina sp. nov., an extremely halophilic archaeon isolated from a salt lake. *Int. J. Syst. Evol. Microbiol.* 58, 2880–2884. doi:10.1099/ijs.0.2008/001602-0.

Gutierrez, M. C., Castillo, A. M., Kamekura, M., Xue, Y., Ma, Y., Cowan, D. A., et al. (2007). Halopiger xanaduensis gen. nov., sp. nov., an extremely halophilic archaeon isolated from saline Lake Shangmatala in Inner Mongolia, China. *Int. J. Syst. Evol. Microbiol.* 57, 1402–1407. doi:10.1099/ijs.0.65001-0.

Gutierrez, M. C., Castillo, A. M., Pagaling, E., Heaphy, S., Kamekura, M., Xue, Y., et al. (2008). Halorubrum kocurii sp. nov., an archaeon isolated from a saline lake. *Int. J. Syst. Evol. Microbiol.* 58, 2031–2035. doi:10.1099/ijs.0.65840-0.

Gutierrez, M. C., Kamekura, M., Holmes, M. L., Dyall-Smith, M. L., and Ventosa, A. (2002). Taxonomic characterization of Haloferax sp. (“H. alicantei”) strain Aa 2.2: Description of Haloferax lucentensis sp. nov. *Extremophiles* 6, 479–483. doi:10.1007/s00792-002-0282-7.

Hafenbradl, D., Keller, M., Dirmeier, R., Rachel, R., Roßnagel, P., Burggraf, S., et al. (1996a). Ferroglobus placidus gen. nov., sp. nov., a novel hyperthermophilic archaeum that oxidizes Fe 2+ at neutral pH under anoxic conditions. *Arch. Microbiol.* 166, 308–314. doi:10.1007/s002030050388.

Hafenbradl, D., Keller, M., and Stetter, K. O. (1996b). Lipid analysis of Methanopyrus kandleri. *FEMS Microbiol. Lett.* 136, 199–202. doi:10.1016/0378-1097(96)84201-7.

Han, D., and Cui, H. L. (2014a). Halobacterium rubrum sp. nov., isolated from a marine solar saltern. *Arch. Microbiol.* 196, 847–851. doi:10.1007/s00203-014-1023-x.

Han, D., and Cui, H. L. (2014b). Haloplanus litoreus sp. nov. and Haloplanus ruber sp. nov., from a marine solar saltern and an aquaculture farm, respectively. *Antonie van Leeuwenhoek, Int. J. Gen. Mol. Microbiol.* 105, 679–685. doi:10.1007/s10482-014-0123-6.

Han, D., and Cui, H. L. (2014c). Halosimplex pelagicum sp. nov. and Halosimplex rubrum sp. nov., isolated from salted brown alga Laminaria, and emended description of the genus Halosimplex. *Int. J. Syst. Evol. Microbiol.* 64, 169–173. doi:10.1099/ijs.0.056887-0.

Han, D., and Cui, H. L. (2015). Halorubrum laminariae sp. nov., isolated from the brine of salted brown alga Laminaria. *Antonie van Leeuwenhoek, Int. J. Gen. Mol. Microbiol.* 107, 217–223. doi:10.1007/s10482-014-0319-9.

Han, D., Cui, H. L., and Li, Z. R. (2014). Halopenitus salinus sp. nov., isolated from the brine of salted brown alga Laminaria. *Antonie van Leeuwenhoek, Int. J. Gen. Mol. Microbiol.* 106, 743–749. doi:10.1007/s10482-014-0244-y.

Han, D., Zhang, W.-J., Cui, H.-L., and Li, Z.-R. (2015). Halovenus rubra sp. nov., Isolated from Salted Brown Alga Laminaria. *Curr. Microbiol.* 70, 91–95. doi:10.1007/s00284-014-0685-6.

Han, D., Zhu, L., and Cui, H. L. (2019). Halorussus litoreus sp. Nov., isolated from the salted brown alga laminaria. *Int. J. Syst. Evol. Microbiol.* 69, 767–772. doi:10.1099/ijsem.0.003233.

Hannington, M. D., de Ronde, C. E. J., and Peterson, S. (2005). Sea-floor tectonics and submarine hydrothermal systems. *Econ. Geol. 100th Anniv. Vol.* 100, 111–141.

Hensel, R., Matussek, K., Michalke, K., Tacke, L., Tindall, B. J., Kohlhoff, M., et al. (1997). Sulfophobococcus zilligii gen. nov., spec, nov. a novel hyperthermophilic archaeum isolated from hot alkaline springs of Iceland. *Syst. Appl. Microbiol.* 20, 102–110. doi:10.1016/S0723-2020(97)80054-9.

Hensley, S. A., Jung, J.-H., Park, C.-S., and Holden, J. F. (2014). Thermococcus paralvinellae sp. nov. and Thermococcus cleftensis sp. nov. of hyperthermophilic heterotrophs from deep-sea hydrothermal vents. *Int. J. Syst. Evol. Microbiol.* 64, 3655–3659. doi:10.1099/ijs.0.066100-0.

Hezayen, F. F., Gutiérrez, M. C., Steinbüchel, A., Tindall, B. J., and Rehm, B. H. A. (2010). Halopiger aswanensis sp. nov., a polymer-producing and extremely halophilic archaeon isolated from hypersaline soil. *Int. J. Syst. Evol. Microbiol.* 60, 633–637. doi:10.1099/ijs.0.013078-0.

Hezayen, F. F., Rehm, B., Tindall, B. J., and Steinbuchel, A. (2001). Transfer of Natrialba asiatica B1T to Natrialba taiwanensis sp. nov. and description of Natrialba aegyptiaca sp. nov., a novel extremely halophilic, aerobic, non-pigmented member of the Archaea from Egypt that produces extracellular poly(glutamic acid). *Int. J. Syst. Evol. Microbiol.* 51, 1133–1142. doi:10.1099/00207713-51-3-1133.

Hezayen, F. F., Tindall, B. J., Steinbüchel, A., and Rehm, B. H. A. (2002). Characterization of a novel halophilic archaeon, Halobiforma haloterrestris gen. nov., sp. nov., and transfer of Natronobacterium nitratireducens to Halobiforma nitratireducens comb. nov. *Int. J. Syst. Evol. Microbiol.* 52, 2271–2280. doi:10.1099/ijs.0.02324-0.

Hou, J., Zhao, Y. J., Zhu, L., and Cui, H. L. (2018). Salinirubellus salinus gen. nov., sp. nov., isolated from a marine solar saltern. *Int. J. Syst. Evol. Microbiol.* 68, 1874–1878. doi:10.1099/ijsem.0.002757.

Hu, L., Pan, H., Xue, Y., Ventosa, A., Cowan, D. A., Jones, B. E., et al. (2008). Halorubrum luteum sp. nov., isolated from Lake Chagannor, Inner Mongolia, China. *Int. J. Syst. Evol. Microbiol.* 58, 1705–1708. doi:10.1099/ijs.0.65700-0.

Huber, G., and Stetter, K. O. (1991). Sulfolobus metallicus, sp. nov., a Novel Strictly Chemolithoautotrophic Thermophilic Archaeal Species of Metal-Mobilizers. *Syst. Appl. Microbiol.* 14, 372–378. doi:10.1016/S0723-2020(11)80312-7.

Huber, H., Burggraf, S., Mayer, T., Wyschkony, I., Rachel, R., and Stetter, K. O. (2000). Ignicoccus gen. nov., a novel genus of hyperthermophilic, chemolithoautotrophic Archaea, represented by two new species, Ignicoccus islandicus sp. nov. and Ignicoccus pacificus sp. nov. *Int. J. Syst. Evol. Microbiol.* 50, 2093–2100. doi:10.1099/00207713-50-6-2093.

Huber, R., Dyba, D., Huber, H., Burggraf, S., and Rachel, R. (1998). Sulfur-inhibited Thermosphaera aggregans sp. nov., a new genus of hyperthermophilic archaea isolated after its prediction from environmentally derived 16S rRNA sequences. *Int. J. Syst. Bacteriol.* 48, 31–38. doi:10.1099/00207713-48-1-31.

Huber, R., Sabine, J. S., Rachel, R., Burggraf, S., Jannasch, H. W., and Stetter, K. O. (1995a). Thermococcus chitonophagus sp. nov., a novel, chitin-degrading, hyperthermophilic archaeum from a deep-sea hydrothermal vent environment. *Arch. Microbiol.* 164, 255–264.

Huber, R., Stöhr, J., Hohenhaus, S., Rachel, R., Burggraf, S., Jannasch, H. W., et al. (1995b). Thermococcus chitonophagus sp. nov., a novel, chitin-degrading, hyperthermophilic archaeum from a deep-sea hydrothermal vent environment. *Arch. Microbiol.* 164, 255–264. doi:10.1007/BF02529959.

Hwang, H.-B., Kim, Y.-E., Koh, H.-W., Song, H. S., Roh, S. W., Kim, S.-J., et al. (2017). Haloplanus salinarum sp. nov., an extremely halophilic archaeon isolated from a solar saltern. *Int. J. Syst. Evol. Microbiol.* 67, 4456–4461. doi:10.1099/ijsem.0.002313.

Ihara, K., Watanabe, S., and Tamura, T. (1997). Two New Extremely Halophilic Archaea Collected in Argentina. *Int. J. Syst. Bacteriol.*, 73–77.

Infante-Domínguez, C., Corral, P., Sánchez-Porro, C., and Ventosa, A. (2015). Halovenus salina sp. nov., an extremely halophilic archaeon isolated from a saltern. *Int. J. Syst. Evol. Microbiol.* 65, 3016–3023. doi:10.1099/ijs.0.000370.

Inoue, K., Itoh, T., Ohkuma, M., and Kogure, K. (2011). Halomarina oriensis gen. nov., sp. nov., a halophilic archaeon isolated from a seawater aquarium. *Int. J. Syst. Evol. Microbiol.* 61, 942–946. doi:10.1099/ijs.0.020677-0.

Ishibashi, J.-I., Ebina, N., Tsutsumi, S., and Kawagucci, S. (2015). Fluid geochemistry of vent fields at submarine arc volcanoes in the Izu-Bonin arc. in *Goldschmidt abstracts*, 1382.

Itoh, T., Suzuki, K., and Nakase, T. (1998). Thermocladium modestius gen. nov., sp. nov., a new genus of rod-shaped , extremely thermophilic crenarchaeote. *Int. J. Syst. Bacteriol.* 48, 879–887. doi:10.1.1.600.9517.

Itoh, T., Suzuki, K., and Nakase, T. (2002). Vulcanisaeta distributa gen. nov., sp. nov., and Vulcanisaeta souniana sp. nov., novel hyperthermophilic, rod-shaped crenarchaeotes isolated from hot springs in Japan. *Int. J. Syst. Evol. Microbiol.* 52, 1097–1104. doi:10.1099/00207713-52-4-1097.

Itoh, T., Suzuki, K., Sanchez, P. C., and Nakase, T. (1999). Caldivirga maquilingensis gen. nov., sp. nov., a new genus of rod-shaped crenarchaeote isolated from a hot spring in the Philippines. *Int. J. Syst. Bacteriol.* 49, 1157–1163.

Itoh, T., Suzuki, K., Sanchez, P. C., and Nakase, T. (2003). Caldisphaera lagunensis gen. nov., sp. nov., a novel thermoacidophilic crenarchaeote isolated from a hot spring at Mt Maquiling, Philippines. *Int. J. Syst. Evol. Microbiol.* 53, 1149–1154. doi:10.1099/ijs.0.02580-0.

Itoh, T., Yamaguchi, T., Zhou, P., and Takashina, T. (2005). Natronolimnobius baerhuensis gen. nov.,

sp. nov. and Natronolimnobius innermongolicus sp. nov., novel haloalkaliphilic archaea isolated from soda lakes in Inner Mongolia, China. *Extremophiles* 9, 111–116. doi:10.1007/s00792-004-0426-z.

Itoh, T., Yoshikawa, N., and Takashina, T. (2007). Thermogymnomonas acidicola gen. nov., sp. nov., a novel thermoacidophilic, cell wall-less archaeon in order Thermoplasmatales, isolated from a solfataric soil in Hakone, Japan. *Int. J. Syst. Evol. Microbiol.* 57, 2557–2561. doi:10.1099/ijs.0.65203-0.

Itoh, Y. H., Kurosawa, N., Uda, I., Sugai, A., Tanoue, S., Itoh, T., et al. (2001). Metallosphaera sedula TA-2, a calditoglycerocaldarchaeol deletion strain of a thermoacidophilic archaeon. *Extremophiles* 5, 241–245. doi:10.1007/s007920100195.

Jahn, U., Summons, R., Sturt, H., Grosjean, E., and Huber, H. (2004). Composition of the lipids of Nanoarchaeum equitans and their origin from its host Ignicoccus sp. strain KIN4/I. *Arch. Microbiol.* 182, 404–413. doi:10.1007/s00203-004-0725-x.

Jan, R.-L., Wu, J., Chaw, S.-M., Tsai, C.-W., and Tsen, S. (1999). A novel species of thermoacidophilic archaeon, Sulfolobus yangmingensis sp. nov. 49, 1809–1816.

Jensen, S. M., Brandl, M., Treusch, A. H., and Ejsing, C. S. (2015). Structural characterization of ether lipids from the archaeon Sulfolobus islandicus by high-resolution shotgun lipidomics. *J. Mass Spectrom.* 50, 476–487. doi:10.1002/jms.3553.

Juez, G., Rodriguez-Valera, F., Ventosa, A., and Kushner, D. J. (1986). Haloarcula hispanica spec. nov. and Haloferax gibbonsii spec, nov., Two New Species of Extremely Halophilic Archaebacteria. *Syst. Appl. Microbiol.* 8, 75–79. doi:10.1016/S0723-2020(86)80152-7.

Jung, M.-Y., Islam, M. A., Gwak, J.-H., Kim, J.-G., and Rhee, S.-K. (2018). Nitrosarchaeum koreense gen. nov., sp. nov., an aerobic and mesophilic, ammonia-oxidizing archaeon member of the phylum Thaumarchaeota isolated from agricultural soil. *Int. J. Syst. Evol. Microbiol.* 68, 3084–3095. doi:10.1099/ijsem.0.002926.

Jung, M.-Y., Kim, J.-G., Sinninghe Damsté, J. S., Rijpstra, W. I. C., Madsen, E. L., Kim, S.-J., et al. (2016). A hydrophobic ammonia-oxidizing archaeon of the Nitrosocosmicus clade isolated from coal tar-contaminated sediment. *Environ. Microbiol. Rep.* 8, 983–992. doi:10.1111/1758-2229.12477.

Jung, M.-Y., Park, S.-J., Kim, S.-J., Kim, J.-G., Sinninghe Damsté, J. S., Jeon, C. O., et al. (2014). A Mesophilic, Autotrophic, Ammonia-Oxidizing Archaeon of Thaumarchaeal Group I.1a Cultivated from a Deep Oligotrophic Soil Horizon. *Appl. Environ. Microbiol.* 80, 3645–3655. doi:10.1128/AEM.03730-13.

Kamagata, Y., Kawasaki, H., Oyaizu, H., Nakamura, K., Mikami, E., Endo, G., et al. (1992). Characterization of Three Thermophilic Strains of Methanothrix (“Methanosaeta”) thermophila sp. nov. and Rejection of Methanothrix (“Methanosaeta”) thermoacetophila. *Int. J. Syst. Bacteriol.* 42, 463–468. doi:10.1099/00207713-42-3-463.

Kamekura, M., Dyall-Smith, M. L., Upasani, V., Ventosa, A., and Kates, M. (1997). Diversity of Alkaliphilic Halobacteria: Proposals for Transfer of Natronobacterium vacuolatum,

Natronobacterium magadii, and Natronobacterium pharaonis to Halorubrum, Natrialba, and Natronomonas gen. nov., Respectively, as Halorubrum vacuolatum comb. nov. *Int. J. Syst. Bacteriol.* 47, 853–857. doi:10.1099/00207713-47-3-853.

Kanai, H., Kobayashi, T., Aono, R., and Kudo, T. (1995). Natronococcus amylolyticus sp. nov., a haloalkaliphilic archaeon. *Int. J. Syst. Bacteriol.* 45, 762–766.

Kharroub, K., Quesada, T., Ferrer, R., Fuentes, S., Aguilera, M., Boulahrouf, A., et al. (2006). Halorubrum ezzemmoulense sp. nov., a halophilic archaeon isolated from Ezzemoul sabkha, Algeria. *Int. J. Syst. Evol. Microbiol.* 56, 1583–1588. doi:10.1099/ijs.0.64272-0.

Khelaifia, S., Caputo, A., Andrieu, C., Cadoret, F., Armstrong, N., Michelle, C., et al. (2018). Genome sequence and description of Haloferax massiliense sp. nov., a new halophilic archaeon isolated from the human gut. *Extremophiles* 22, 485–498. doi:10.1007/s00792-018-1011-1.

Kim, K. K., Lee, K. C., and Lee, J. S. (2011). Halogranum salarium sp. nov., a halophilic archaeon isolated from sea salt. *Syst. Appl. Microbiol.* 34, 576–580. doi:10.1016/j.syapm.2011.03.007.

Kim, T. Y., Kim, S. J., Park, S. J., Kim, J. G., Cha, I. T., Jung, M. Y., et al. (2013). Natronomonas gomsonensis Sp. Nov., isolated from a solar saltern. *Antonie van Leeuwenhoek, Int. J. Gen. Mol. Microbiol.* 104, 627–635. doi:10.1007/s10482-013-9970-9.

Knappy, C. S., Nunn, C. E. M., Morgan, H. W., and Keely, B. J. (2011). The major lipid cores of the archaeon Ignisphaera aggregans: Implications for the phylogeny and biosynthesis of glycerol monoalkyl glycerol tetraether isoprenoid lipids. *Extremophiles* 15, 517–528. doi:10.1007/s00792-011-0382-3.

Kochetkova, T. V., Kublanov, I. V., Toshchakov, S. V., Osburn, M. R., Novikov, A. A., Bonch-Osmolovskaya, E. A., et al. (2016). Thermogladius calderae gen. nov., sp. nov., an anaerobic, hyperthermophilic crenarchaeote from a Kamchatka hot spring. *Int. J. Syst. Evol. Microbiol.* 66, 1407–1412. doi:10.1099/ijsem.0.000916.

Koga, Y., Akagawa-Matsushita, M., Ohga, M., and Nishihara, M. (1993). Taxonomic Significance of the Distribution of Component Parts of Polar Ether Lipids in Methanogens. *Syst. Appl. Microbiol.* 16, 342–351. doi:10.1016/S0723-2020(11)80264-X.

Koga, Y., Morii, H., Akagawa-Matsushita, M., and Ohga, M. (1998). Correlation of polar lipid composition with 16S rRNA phylogeny in methanogens. Further analysis of lipid component parts. *Biosci. Biotechnol. Biochem.* 62, 230–236. doi:10.1271/bbb.62.230.

Koga, Y., Ohga, M., Nishihara, M., and Morii, H. (1987). Distribution of a diphytanyl ether analog of phosphatidylserine and an ethanolamine-containing tetraether lipid in methanogenic bacteria. *Syst. Appl. Microbiol.* 9, 176–182. doi:10.1016/S0723-2020(87)80019-X.

Koh, H.-W., Park, S.-J., Song, U., Song, H. S., Roh, S. W., and Yim, K. J. (2015). Halolamina sediminis sp. nov., an extremely halophilic archaeon isolated from solar salt. *Int. J. Syst. Evol. Microbiol.* 65, 2479–2484. doi:10.1099/ijs.0.000287.

Kondo, Y., Minegishi, H., Echigo, A., Shimane, Y., Kamekura, M., Itoh, T., et al. (2015). Halorubrum gandharaense sp. nov an alkaliphilic haloarchaeon from commercial rock salt. *Int. J. Syst. Evol.*

Microbiol. 65, 2345–2350. doi:10.1099/ijs.0.000261.

Kondo, Y., Minegishi, H., Echigo, A., Shimane, Y., Kamekura, M., Itoh, T., et al. (2016). Haloparvum alkalitolerans sp. Nov., alkali-tolerant haloarchaeon isolated from commercial salt. *Int. J. Syst. Evol. Microbiol.* 66, 5314–5319. doi:10.1099/ijsem.0.001513.

Kuwabara, T., Minaba, M., Iwayama, Y., Inouye, I., Nakashima, M., Marumo, K., et al. (2005). Thermococcus coalescens sp. nov., a cell-fusing hyperthermophilic archaeon from Suiyo Seamount. *Int. J. Syst. Evol. Microbiol.* 55, 2507–2514. doi:10.1099/ijs.0.63432-0.

Kuwabara, T., Minaba, M., Ogi, N., and Kamekura, M. (2007). Thermococcus celericrescens sp. nov., a fast-growing and cell-fusing hyperthermophilic archaeon from a deep-sea hydrothermal vent. *Int. J. Syst. Evol. Microbiol.* 57, 437–443. doi:10.1099/ijs.0.64597-0.

Lai, D., Springstead, J. R., and Monbouquette, H. G. (2008). Effect of growth temperature on ether lipid biochemistry in Archaeoglobus fulgidus. *Extremophiles* 12, 271–278. doi:10.1007/s00792-007-0126-6.

Langworthy, T. A., and Pond, L. (1986). Archaebacterial ether lipids and chemotaxonomy. *Syst. Appl. Microbiol.* 7, 253–257. doi:10.1016/S0723-2020(86)80015-7.

Lanzotti, V., Rosa, M. De, Trincone, A., Basso, A. L., Gambacorta, A., and Zillig, W. (1987). Complex lipids from Desulfurococcus mobilis , a sulfur-reducing archaebacterium Virginia Lanzotti Agata Gambacorta a and Wolfram Zillig '. 922, 95–102.

Lanzotti, V., Trincone, A., Nicolaus, B., Zillig, W., Rosa, M. De, and Gambacorta, A. (1989). Complex lipids of Pyrococcus and AN1, thermophilic members of archaebcteria belonging to Thermococcales. *Biochim. Biophys. Acta (BBA)/Lipids Lipid Metab.* 1004, 44–48. doi:10.1016/0005-2760(89)90210-5.

Lattuati, A., Guezennec, J., Metzger, P., and Largeau, C. (1998). Lipids of Thermococcus hydrothermalis, an archaea isolated from a deep- sea hydrothermal vent. *Lipids* 33, 319–326. doi:10.1007/s11745-998-0211-0.

Lauerer, G., Kristjansson, J. K., Langworthy, T. A., König, H., and Stetter, K. O. (1986). Methanothermus sociabilis sp. nov., a Second Species within the Methanothermaceae Growing at 97°C. *Syst. Appl. Microbiol.* 8, 100–105. doi:10.1016/S0723-2020(86)80156-4.

Lê, S., Josse, J., Rennes, A., and Husson, F. (2008). FactoMineR: An R package for multivariate analysis. Available at: http://www.jstatsoft.org/.

Li, Y., Zhou, Y., Lü, Z. Z., and Cui, H. L. (2018). Halobium Salinum sp. nov., Isolated from a Marine Solar Saltern. *Curr. Microbiol.* 75, 6–10. doi:10.1007/s00284-017-1343-6.

Liu, B. B., Tang, S. K., Cui, H. L., Zhang, Y. G., Li, L., Zhang, Y. M., et al. (2013a). Halopelagius fulvigenes sp. nov., a halophilic archaeon isolated from a lake. *Int. J. Syst. Evol. Microbiol.* 63, 2192–2196. doi:10.1099/ijs.0.045773-0.

Liu, B. B., Tang, S. K., Zhang, Y. G., Lu, X. H., Li, L., Cheng, J., et al. (2013b). Halalkalicoccus paucihalophilus sp. nov., a halophilic archaeon from Lop Nur region in Xinjiang, northwest of

China. *Antonie van Leeuwenhoek, Int. J. Gen. Mol. Microbiol.* 103, 1007–1014. doi:10.1007/s10482-013-9880-x.

Liu, B. B., Zhao, W. Y., Chu, X., Hozzein, W. N., Prabhu, D. M., Wadaan, M. A. M., et al. (2014). Haladaptatus pallidirubidus sp. nov., a halophilic archaeon isolated from saline soil samples in Yunnan and Xinjiang, China. *Antonie van Leeuwenhoek, Int. J. Gen. Mol. Microbiol.* 106, 901–910. doi:10.1007/s10482-014-0259-4.

Liu, Q., Ren, M., and Zhang, L. L. (2015). Natribaculum breve gen. Nov., sp. nov. and natribaculum longum sp. nov., Halophilic archaea isolated from saline soil. *Int. J. Syst. Evol. Microbiol.* 65, 604–608. doi:10.1099/ijs.0.060541-0.

Liu, Y., Osinski, T., Wang, F., Krupovic, M., Schouten, S., Kasson, P., et al. (2018). Structural conservation in a membrane-enveloped filamentous virus infecting a hyperthermophilic acidophile. *Nat. Commun.* 9, 2–11. doi:10.1038/s41467-018-05684-6.

Lizama, C., Monteoliva-Sánchez, M., Suárez-García, A., Roselló-Mora, R., Aguilera, M., and Campos, VictorianoRamos-Cormenzana, A. (2002). Halorubrum tebenquichense sp. nov., a novel halophilic archaeon isolated from the Atacama Saltern, Chile. *Int. J. Syst. Evol. Microbiol.* 52, 149–155. doi:10.1099/00207713-52-1-149.

Lü, Z. Z., Li, Y., Zhou, Y., Cui, H. L., and Li, Z. R. (2017). Halobacterium litoreum sp. Nov., isolated from a marine solarsaltern. *Int. J. Syst. Evol. Microbiol.* 67, 4095–4099. doi:10.1099/ijsem.0.002259.

Macalady, J. L., Vestling, M. M., Baumler, D., Boekelheide, N., Kaspar, C. W., and Banfield, J. F. (2004). Tetraether-linked membrane monolayers in Ferroplasma spp: a key to survival in acid. *Extremophiles* 8, 411–419. doi:10.1007/s00792-004-0404-5.

Makhdoumi-Kakhki, A., Amoozegar, M. A., Bagheri, M., Ramezani, M., and Ventosa, A. (2012a). Haloarchaeobius iranensis gen. nov., sp. nov., an extremely halophilic archaeon isolated from a saline lake. *Int. J. Syst. Evol. Microbiol.* 62, 1021–1026. doi:10.1099/ijs.0.033167-0.

Makhdoumi-Kakhki, A., Amoozegar, M. A., and Ventosa, A. (2012b). Halovenus aranensis gen. nov., sp. nov., an extremely halophilic archaeon from Aran-Bidgol salt lake. *Int. J. Syst. Evol. Microbiol.* 62, 1331–1336. doi:10.1099/ijs.0.031419-0.

Mancinelli, R. L., Landheim, R., Sanchez-Porro, C., Dornmayr-Pfaffenhuemer, M., Gruber, C., Legat, A., et al. (2009). Halorubrum chaoviator sp. nov., a haloarchaeon isolated from sea salt in Baja California, Mexico, Western Australia and Naxos, Greece. *Int. J. Syst. Evol. Microbiol.* 59, 1908–1913. doi:10.1099/ijs.0.000463-0.

Marteinsson, V. T., Birrien, J., Reysenbach, A., Vernet, M., Marie, D., Gamba, A., et al. (1999). Thermococcus barophilus sp. nov., a new barophilic and hyperthermophilic archaeon isolated under high hydrostatic pressure from a deep-sea hydrothermal vent. *Int. J. Syst. Bacteriol.* 49, 351–359. doi:doi: 10.1099/00207713-49-2-351.

Matsuno, Y., Sugai, A., Higashibata, H., Fukuda, W., Ueda, K., Uda, I., et al. (2009). Effect of growth temperature and growth phase on the lipid composition of the archaeal membrane from Thermococcus kodakaraensis. *Biosci. Biotechnol. Biochem.* 73, 104–108.

doi:10.1271/bbb.80520.

McDuff, S., King, G. M., Neupane, S., and Myers, M. R. (2016). Isolation and characterization of extremely halophilic CO-oxidizing Euryarchaeota from hypersaline cinders, sediments and soils and description of a novel CO oxidizer, Haloferax namakaokahaiae Mke2.3T, sp. nov. *FEMS Microbiol. Ecol.* 92, 1–8. doi:10.1093/femsec/fiw028.

McGenity, T. J., Gemmell, R. T., and Grant, W. D. (1998). Proposal of a new halobacterial genus Natrinema gen. nov., with two species Natrinema pellirubrum nom. nov. and Natrinema pallidum nom. nov. *Int. J. Syst. Bacteriol.* 48, 1187–1196. doi:10.1099/00207713-48-4-1187.

Meador, T. B., Gagen, E. J., Loscar, M. E., Goldhammer, T., Yoshinaga, M. Y., Wendt, J., et al. (2014). Thermococcus kodakarensis modulates its polar membrane lipids and elemental composition according to growth stage and phosphate availability. *Front. Microbiol.* 5, 1–13. doi:10.3389/fmicb.2014.00010.

Mehrshad, M., Amoozegar, M. A., Makhdoumi, A., Fazeli, S. A. S., Farahani, H., Asadi, B., et al. (2016). Halosiccatus urmianus gen. nov., sp. nov., a haloarchaeon from a salt lake. *Int. J. Syst. Evol. Microbiol.* 66, 725–730. doi:10.1099/ijsem.0.000781.

Mehrshad, M., Amoozegar, M. A., Makhdoumi, A., Rasooli, M., Asadi, B., Schumann, P., et al. (2015). Halovarius luteus gen. nov., sp. nov., an extremely halophilic archaeon from a salt lake. *Int. J. Syst. Evol. Microbiol.* 65, 2420–2425. doi:10.1099/ijs.0.000279.

Minegishi, H., Echigo, A., Nagaoka, S., Kamekura, M., and Usami, R. (2010). Halarchaeum acidiphilum gen. nov., sp. nov., a moderately acidophilic haloarchaeon isolated from commercial solar salt. *Int. J. Syst. Evol. Microbiol.* 60, 2513–2516. doi:10.1099/ijs.0.013722-0.

Minegishi, H., Echigo, A., Shimane, Y., Kamekura, M., Itoh, T., Ohkuma, M., et al. (2015a). Halococcus agarilyticus sp. nov., an agar-degrading haloarchaeon isolated from commercial salt. *Int. J. Syst. Evol. Microbiol.* 65, 1634–1639. doi:10.1099/ijs.0.000151.

Minegishi, H., Enomoto, S., Echigo, A., Shimane, Y., Kondo, Y., Inoma, A., et al. (2017). Salinarchaeum chitinilyticum sp. nov., a chitin-degrading haloarchaeon isolated from commercial salt. *Int. J. Syst. Evol. Microbiol.* 67, 2274–2278. doi:10.1099/ijsem.0.001941.

Minegishi, H., Kuwahara, A., Usami, R., Itoh, T., Echigo, A., Kamekura, M., et al. (2015b). Halocalculus aciditolerans gen. nov., sp. nov., an acid-tolerant haloarchaeon isolated from commercial salt. *Int. J. Syst. Evol. Microbiol.* 65, 1640–1645. doi:10.1099/ijs.0.000152.

Minegishi, H., Shimogaki, R., Enomoto, S., Echigo, A., Kondo, Y., Nagaoka, S., et al. (2016). Halopiger thermotolerans sp. Nov., a thermotolerant haloarchaeon isolated from commercial salt. *Int. J. Syst. Evol. Microbiol.* 66, 4975–4980. doi:10.1099/ijsem.0.001455.

Minegishi, H., Yamauchi, Y., Echigo, A., Shimane, Y., Kamekura, M., Itoh, T., et al. (2013). Halarchaeum nitratireducens sp. nov., a moderately acidophilic haloarchaeon isolated from commercial sea salt. *Int. J. Syst. Evol. Microbiol.* 63, 4202–4206. doi:10.1099/ijs.0.054668-0.

Montalvo-Rodriguez, R., Lopez-Garriga, J., Vreeland, R. H., Oren, A., Ventosa, A., and Kamekura, M. (2000). Haloterrigena thermotolerans sp. nov., a halophilic archaeon from Puerto Rico. *Int.*

J. Syst. Evol. Microbiol. 50, 1065–1071. doi:10.1099/00207713-50-3-1065.

Montalvo-Rodriguez, R., Vreeland, R. H., Oren, A., Kessel, M., Betancourt, C., and Lopez-Garriga, J. (1998). Halogeometricum borinquense gen. nov., sp. nov., a novel halophilic archaeon from Puerto Rico. *Int. J. Syst. Bacteriol.* 48, 1305–1312. doi:10.1099/00207713-48-4-1305.

Montero, C. G., Ventosa, A., Rodriguez-Valera, F., Kates, M., Moldoveanu, N., and Ruiz-Berraquero, F. (1989). Halococcus saccharolyticus sp. nov., a New Species of Extremely Halophilic Non-alkaliphilic Cocci. *Syst. Appl. Microbiol.* 12, 167–171. doi:10.1016/S0723-2020(89)80010-4.

Mori, K., Nurcahyanto, D. A., Kawasaki, H., Lisdiyanti, P., Yopi, and Suzuki, K. I. (2016a). Haloarchaeobius baliensis sp. Nov., isolated from a solar saltern. *Int. J. Syst. Evol. Microbiol.* 66, 38–43. doi:10.1099/ijsem.0.000672.

Mori, K., Nurcahyanto, D. A., Kawasaki, H., Lisdiyanti, P., Yopi, and Suzuki, K. I. (2016b). Halobium palmae gen. Nov., sp. nov., an extremely halophilic archaeon isolated from a solar saltern. *Int. J. Syst. Evol. Microbiol.* 66, 3799–3804. doi:10.1099/ijsem.0.001267.

Morii, H., Yagi, H., Akutsu, H., Nomura, N., Sako, Y., and Koga, Y. (1999). A novel phosphoglycolipid archaetidyl(glucosyl)inositol with two sesterterpanyl chains from the aerobic hyperthermophilic archaeon Aeropyrum pernix K1. *Biochim. Biophys. Acta - Mol. Cell Biol. Lipids* 1436, 426–436. doi:10.1016/S0005-2760(98)00157-X.

Morikawa, M., Izawa, Y., Rashid, N., Hoaki, T., and Imanaka, T. (1994). Purification and characterization of a thermostable thiol protease from a newly isolated hyperthermophilic Pyrococcus sp. *Appl. Environ. Microbiol.* 60, 4559–66. Available at: http://www.ncbi.nlm.nih.gov/pubmed/7811092.

Mou, Y. Z., Qiu, X. X., Zhao, M. L., Cui, H. L., Oh, D., and Dyall-Smith, M. L. (2012). Halohasta litorea gen. nov. sp. nov., and Halohasta litchfieldiae sp. nov., isolated from the Daliang aquaculture farm, China and from Deep Lake, Antarctica, respectively. *Extremophiles* 16, 895–901. doi:10.1007/s00792-012-0485-5.

Mwatha, W. E., and Grant, W. D. (1993). Natronobacterium vacuolata sp. nov., a Haloalkaliphilic Archaeon Isolated from Lake Magadi, Kenya. *Int. J. Syst. Bacteriol.* 43, 401–404. doi:10.1099/00207713-43-3-401.

Nagaoka, S., Minegishi, H., Echigo, A., Shimane, Y., Kamekura, M., and Usami, R. (2011). Halostagnicola alkaliphila sp. nov., an alkaliphilic haloarchaeon from commercial rock salt. *Int. J. Syst. Evol. Microbiol.* 61, 1149–1152. doi:10.1099/ijs.0.023119-0.

Nagaoka, S., Minegishi, H., Echigo, A., and Usami, R. (2010). Halostagnicola kamekurae sp. nov., an extremely halophilic archaeon from solar salt. *Int. J. Syst. Evol. Microbiol.* 60, 2828–2831. doi:10.1099/ijs.0.014449-0.

Naghoni, A., Emtiazi, G., Amoozegar, M. A., Rasooli, M., Etemadifar, Z., Shahzadeh Fazeli, S. A., et al. (2017). Natronoarchaeum persicum sp. nov., a haloarchaeon isolated from a hypersaline lake. *Int. J. Syst. Evol. Microbiol.* 67, 3339–3344. doi:10.1099/ijsem.0.002116.

Nakamura, K., Takahashi, A., Mori, C., Tamaki, H., Mochimaru, H., Nakamura, K., et al. (2013).

Methanothermobacter tenebrarum sp. nov., a hydrogenotrophic, thermophilic methanogen isolated from gas-associated formation water of a natural gas field. *Int. J. Syst. Evol. Microbiol.* 63, 715–722. doi:10.1099/ijs.0.041681-0.

Namwong, S., Tanasupawat, S., Kudo, T., and Itoh, T. (2011). Haloarcula salaria sp. nov. and Haloarcula tradensis sp. nov., isolated from salt in Thai fish sauce. *Int. J. Syst. Evol. Microbiol.* 61, 231–236. doi:10.1099/ijs.0.021790-0.

Namwong, S., Tanasupawat, S., Visessanguan, W., Kudo, T., and Itoh, T. (2007). Halococcus thailandensis sp. nov., from fish sauce in Thailand. *Int. J. Syst. Evol. Microbiol.* 57, 2199–2203. doi:10.1099/ijs.0.65218-0.

Nichols, D. S., Miller, M. R., Davies, N. W., Goodchild, A., Raftery, M., and Cavicchioli, R. (2004). Cold adaptation in the Antarctic archaeon Methanococcoides burtonii involves membrane lipids unsaturation. *J. Bacteriol.* 186, 12. doi:10.1128/JB.186.24.8508–8515.2004.

Nishihara, M., and Koga, Y. (1987). Extraction and composition of polar lipids from the archaebacterium, Methanobacterium thermoautotrophicum: Effective extraction of tetraether lipids by an acidified solvent. *J. Biochem.* 101, 997–1005. doi:10.1093/oxfordjournals.jbchem.a121969.

Oren, A., Elevi, R., Watanabe, S., Ihara, K., and Corcelli, A. (2002). Halomicrobium mukohataei gen. nov., comb. nov., and emended description of Halomicrobium mukohataei. *Int. J. Syst. Evol. Microbiol.* 52, 1831–1835. doi:10.1099/ijs.0.02156-0.

Oren, A., Gurevich, P., Gemmel, R. T., and Teske, A. (1995). Halobaculum gomorrense gen. nov., sp. nov., a Novel Extremely Halophilic Archaeon from the Dead Sea. *Int. J. Syst. Bacteriol.* 45, 747–754. doi:10.1099/00207713-45-4-747.

Oren, A., Ventosa, A., Gutierrez, M. C., and Kamekura, M. (1999). Haloarcula quadrata sp. nov., a square, motile archaeon isolated from a brine pool in Sinai (Egypt). *Int. J. Syst. Bacteriol.* 49, 1149–1155. doi:10.1099/00207713-49-3-1149.

Patell, G., and Sprott, G. (1990). Methanosaeta concilii gen. nov. sp. nov. ("Methanothrix concilii") and Methanosaeta thermoacetophila nom. rev., comb. nov. *Int. J. Syst. Bacteriol.* 40, 79–82.

Pearson, A., Pi, Y., Zhao, W., Li, W., Li, Y., Inskeep, W., et al. (2008). Factors controlling the distribution of archaeal tetraethers in terrestrial hot springs. *Appl. Environ. Microbiol.* 74, 3523–3532. doi:10.1128/aem.02450-07.

Peng, T. J., Liu, L. J., Liu, C., Yang, Z. F., Liu, S. J., and Jiang, C. Y. (2015). Metallosphaera tengchongensis sp. Nov., An acidothermophilic archaeon isolated from a hot spring. *Int. J. Syst. Evol. Microbiol.* 65, 537–542. doi:10.1099/ijs.0.070870-0.

Perevalova, A. A., Bidzhieva, S. K., Kublanov, I. V., Hinrichs, K.-U., Liu, X. L., Mardanov, A. V., et al. (2010). Fervidicoccus fontis gen. nov., sp. nov., an anaerobic, thermophilic crenarchaeote from terrestrial hot springs, and proposal of Fervidicoccaceae fam. nov. and Fervidicoccales ord. nov. *Int. J. Syst. Evol. Microbiol.* 60, 2082–2088. doi:10.1099/ijs.0.019042-0.

Pérez-Davó, A., Aguilera, M., González-Paredes, A., Jiménez-Pranteda, M. L., and Monteoliva-

Sánchez, M. (2015). Halobellus ramosii sp. Nov., an extremely halophilic archaeon isolated from a saline-wetland wildfowl reserve. *Int. J. Syst. Evol. Microbiol.* 65, 3847–3852. doi:10.1099/ijsem.0.000503.

Pesenti, P. T., Sikaroodi, M., Gillevet, P. M., Sánchez-Porro, C., Ventosa, A., and Litchfield, C. D. (2008). Halorubrum californiense sp. nov., an extreme archaeal halophile isolated from a crystallizer pond at a solar salt plant in California, USA. *Int. J. Syst. Evol. Microbiol.* 58, 2710–2715. doi:10.1099/ijs.0.2008/002410-0.

Pley, U., Schipka, J., Gambacorta, A., Jannasch, H. W., Fricke, H., Rachel, R., et al. (1991). Pyrodictium abyssi sp. nov. Represents a Novel Heterotrophic Marine Archaeal Hyperthermophile Growing at 110°C. *Syst. Appl. Microbiol.* 14, 245–253. doi:10.1016/s0723-2020(11)80376-0.

Plumb, J. J., Haddad, C. M., Gibson, J. A. E., and Franzmann, P. D. (2007). Acidianus sulfidivorans sp. nov., an extremely acidophilic, thermophilic archaeon isolated from a solfatara on Lihir Island, Papua New Guinea, and emendation of the genus description. *Int. J. Syst. Evol. Microbiol.* 57, 1418–1423. doi:10.1099/ijs.0.64846-0.

Pope, J. G., McConchie, D. M., Clark, M. D., and Brown, K. L. (2004). Diurnal variations in the chemistry of geothermal fluids after discharge, Champagne Pool, Waiotapu, New Zealand. *Chem. Geol.* 203, 253–272. doi:10.1016/j.chemgeo.2003.10.004.

Prokofeva, M. I., Kostrikina, N. A., Kolganova, T. V., Tourova, T. P., Lysenko, A. M., Lebedinsky, A. V., et al. (2009). Isolation of the anaerobic thermoacidophilic crenarchaeote Acidilobus saccharovorans sp. nov. and proposal of Acidilobales ord. nov., including Acidilobaceae fam. nov. and Caldisphaeraceae fam. nov. *Int. J. Syst. Evol. Microbiol.* 59, 3116–3122. doi:10.1099/ijs.0.010355-0.

Qiu, X.-X., Cui, H.-L., Dyall-Smith, M. L., Han, D., Zhao, M.-L., and Zhang, W.-J. (2013a). Taxonomic study of the genera Halogeometricum and Halosarcina: transfer of Halosarcina limi and Halosarcina pallida to the genus Halogeometricum as Halogeometricum limi comb. nov. and Halogeometricum pallidum comb. nov., respectively. *Int. J. Syst. Evol. Microbiol.* 63, 3915–3919. doi:10.1099/ijs.0.055038-0.

Qiu, X.-X., Mou, Y.-Z., Zhao, M.-L., Zhang, W.-J., Han, D., Ren, M., et al. (2013b). Halobellus inordinatus sp. nov., from a marine solar saltern and an inland salt lake of China. *Int. J. Syst. Evol. Microbiol.* 63, 3975–3980. doi:10.1099/ijs.0.053066-0.

Qiu, X. X., Zhao, M. L., and Cui, H. L. (2014). Natronoarchaeum rubrum sp. nov., isolated from a marine solar saltern, and emended description of the genus Natronoarchaeum. *Int. J. Syst. Evol. Microbiol.* 64, 952–956. doi:10.1099/ijs.0.059071-0.

Qiu, X. X., Zhao, M. L., Han, D., Zhang, W. J., and Cui, H. L. (2013c). Halorubrum rubrum Sp. Nov., an extremely halophilic archaeon from a Chinese salt lake. *Antonie van Leeuwenhoek, Int. J. Gen. Mol. Microbiol.* 104, 885–891. doi:10.1007/s10482-013-0002-6.

Qiu, X., Zhao, M.-L., Han, D., Zhang, W.-J., and Cui, H.-L. (2013d). Haloplanus salinus sp. nov., an extremely halophilic archaeon from a Chinese marine solar saltern. *Arch. Microbiol.* 195, 799–803. doi:10.1007/s00203-013-0929-z.

Rasooli, M., Naghoni, A., Amoozegar, M. A., Mirfeizi, L., Moshtaghi Nikou, M., Shahzadeh Fazeli, S. A., et al. (2017). Natrinema soli sp. nov., a novel halophilic archaeon isolated from a hypersaline wetland. *Int. J. Syst. Evol. Microbiol.* 67, 2142–2147. doi:10.1099/ijsem.0.001909.

Rodriguez-Valera, F., Juez, G., and Kushner, D. J. (1983). Halobacterium mediterranei spec, nov., a New Carbohydrate-Utilizing Extreme Halophile. *Syst. Appl. Microbiol.* 4, 369–381. doi:10.1016/S0723-2020(83)80021-6.

Rogers, K. L., Amend, J. P., and Gurrieri, S. (2007). Temporal changes in fluid chemistry and energy profiles in the Vulcano Island hydrothermal system. *Astrobiology* 7, 905–932. doi:10.1089/ast.2007.0128.

Roh, S. W., and Bae, J. W. (2009). Halorubrum cibi sp. nov., an extremely halophilic archaeon from salt-fermented seafood. *J. Microbiol.* 47, 162–166. doi:10.1007/s12275-009-0016-y.

Roh, S. W., Lee, M. L., and Bae, J. W. (2010). Haladaptatus cibarius sp. nov., an extremely halophilic archaeon from seafood, and emended description of the genus Haladaptatus. *Int. J. Syst. Evol. Microbiol.* 60, 1187–1190. doi:10.1099/ijs.0.013037-0.

Roh, S. W., Nam, Y. Do, Chang, H. W., Sung, Y., Kim, K. H., Lee, H. J., et al. (2007a). Natronococcus jeotgali sp. nov., a halophilic archaeon isolated from shrimp jeotgal, a traditional fermented seafood from Korea. *Int. J. Syst. Evol. Microbiol.* 57, 2129–2131. doi:10.1099/ijs.0.65120-0.

Roh, S. W., Nam, Y. Do, Chang, H. W., Sung, Y., Kim, K. H., Oh, H. M., et al. (2007b). Halalkalicoccus jeotgali sp. nov., a halophilic archaeon from shrimp jeotgal, a traditional Korean fermented seafood. *Int. J. Syst. Evol. Microbiol.* 57, 2296–2298. doi:10.1099/ijs.0.65121-0.

Roh, S. W., Nam, Y.-D., Chang, H.-W., Kim, K.-H., Sung, Y., Kim, M.-S., et al. (2009). Haloterrigena jeotgali sp. nov., an extremely halophilic archaeon from salt-fermented food. *Int. J. Syst. Evol. Microbiol.* 59, 2359–2363. doi:10.1099/ijs.0.008243-0.

Romano, I., Poli, A., Finore, I., Huertas, F. J., Gambacorta, A., Pelliccione, S., et al. (2007). Haloterrigena hispanica sp. nov., an extremely halophilic archaeon from Fuente de Piedra, southern Spain. *Int. J. Syst. Evol. Microbiol.* 57, 1499–1503. doi:10.1099/ijs.0.64895-0.

Ruiz-Romero, E., Sanchez-Lopez, K. B., Coutino-Coutino, M. d. l. A., Gonzalez-Pozos, S., Bello-Lopez, J. M., Lopez-Ramirez, M. P., et al. (2013a). Natronobacterium texcoconense sp. nov., a haloalkaliphilic archaeon isolated from soil of a former lake. *Int. J. Syst. Evol. Microbiol.* 63, 4163–4166. doi:10.1099/ijs.0.053629-0.

Ruiz-Romero, E., Valenzuela-Encinas, C., López-Ramírez, M. P., De Los Angeles Coutiño-Coutiño, M., Marsch, R., and Dendooven, L. (2013b). Natronorubrum texcoconense sp. nov., a haloalkaliphilic archaeon isolated from soil of the former lake Texcoco (Mexico). *Arch. Microbiol.* 195, 145–151. doi:10.1007/s00203-012-0852-8.

Sakai, H. D., and Kurosawa, N. (2017). Sulfodiicoccus acidiphilus gen. nov., sp. nov., a sulfur-inhibited thermoacidophilic archaeon belonging to the order Sulfolobales isolated from a terrestrial acidic hot spring. *Int. J. Syst. Evol. Microbiol.* 67, 1880–1886. doi:10.1099/ijsem.0.001881.

Sako, Y., Nomura, N., Uchida, A., Ishida, Y., Morii, H., Koga, Y., et al. (1996). Aeropyrum pernix gen. nov., sp. nov., a novel aerobic hyperthermophilic archaeon growing at temperatures up to 100 °C. *Int. J. Syst. Bacteriol.* 46, 1070–1077. doi:10.1099/00207713-46-4-1070.

Saralov, A. I., Baslerov, R. V., and Kuznetsov, B. B. (2013). Haloferax chudinovii sp. nov., a halophilic archaeon from Permian potassium salt deposits. *Extremophiles* 17, 499–504. doi:10.1007/s00792-013-0534-8.

Saralov, A. I., Baslerov, R. V., Reutskikh, E. M., and Kuznetsov, B. B. (2012). Halarchaeum solikamskense sp. nov., a thermotolerant neutrophilic haloarchaeon from the foamy products of flotation enrichment of potassium minerals. *Microbiology* 81, 589–595. doi:10.1134/s0026261712050141.

Savage, K. N., Krumholz, L. R., Oren, A., and Elsahed, M. S. (2007). Haladaptatus paucihalophilus gen. nov., sp. nov., a halophilic archaeon isolated from a low-salt, sulfide-rich spring. *Int. J. Syst. Evol. Microbiol.* 57, 19–24. doi:10.1099/ijs.0.64464-0.

Schirmack, J., Mangelsdorf, K., Ganzert, L., Sand, W., Hillebrand-Voiculescu, A., and Wagner, D. (2014). Methanobacterium movilense sp. nov., a hydrogenotrophic, secondary-alcohol-utilizing methanogen from the anoxic sediment of a subsurface lake. *Int. J. Syst. Evol. Microbiol.* 64, 522–527. doi:10.1099/ijs.0.057224-0.

Schleper, C., Puehler, G., Holz, I., Gambacorta, A., Janekovic, D., Santarius, U., et al. (1995). Picrophilus gen. nov., fam. nov.: a novel aerobic, heterotrophic, thermoacidophilic genus and family comprising archaea capable of growth around pH 0. *J. Bacteriol.* 177, 7050–7059. doi:10.1128/jb.177.24.7050-7059.1995.

Schouten, S., Baas, M., Hopmans, E. C., Reysenbach, A.-L., and Damsté, J. S. S. (2008). Tetraether membrane lipids of Candidatus “Aciduliprofundum boonei”, a cultivated obligate thermoacidophilic euryarchaeote from deep-sea hydrothermal vents. *Extremophiles* 12, 119–124. doi:10.1007/s00792-007-0111-0.

Schouten, S., Van Der Meer, M. T. J., Hopmans, E. C., Rijpstra, W. I. C., Reysenbach, A. L., Ward, D. M., et al. (2007). Archaeal and bacterial glycerol dialkyl glycerol tetraether lipids in hot springs of Yellowstone National Park. *Appl. Environ. Microbiol.* 73, 6181–6191. doi:10.1128/AEM.00630-07.

Segerer, A. H., Trincone, A., Gahrtz, M., and Stetter, K. O. (1991). Stygiolobus azoricus gen. nov., sp. nov. represents a novel genus of anaerobic, extremely thermoacidophilic archaebacteria of the order Sulfolobales. *Int. J. Syst. Bacteriol.* 41, 495–501. doi:10.1099/00207713-41-4-495.

Shimada, H., Nemoto, N., Shida, Y., Oshima, T., and Yamagishi, A. (2002). Complete Polar Lipid Composition of Thermoplasma acidophilum HO-62 Determined by High-Performance Liquid Chromatography with Evaporative Light-Scattering Detection. *J. Bacteriol.* 184, 556–563. doi:10.1128/JB.184.2.556-563.2002.

Shimane, Y., Hatada, Y., Minegishi, H., Echigo, A., Nagaoka, S., Miyazaki, M., et al. (2011). Salarchaeum japonicum gen. nov., sp. nov., an aerobic, extremely halophilic member of the Archaea isolated from commercial salt. *Int. J. Syst. Evol. Microbiol.* 61, 2266–2270. doi:10.1099/ijs.0.025064-0.

Shimane, Y., Hatada, Y., Minegishi, H., Mizuki, T., Echigo, A., Miyazaki, M., et al. (2010). Natronoarchaeum mannanilyticum gen. nov., sp. nov., an aerobic, extremely halophilic archaeon isolated from commercial salt. *Int. J. Syst. Evol. Microbiol.* 60, 2529–2534. doi:10.1099/ijs.0.016600-0.

Shimane, Y., Minegishi, H., Echigo, A., Kamekura, M., Itoh, T., Ohkuma, M., et al. (2015). Halarchaeum grantii sp. nov., a moderately acidophilic haloarchaeon isolated from a commercial salt sample. *Int. J. Syst. Evol. Microbiol.* 65, 3830–3835. doi:10.1099/ijsem.0.000501.

Shimane, Y., Nagaoka, S., Minegishi, H., Kamekura, M., Echigo, A., Hatada, Y., et al. (2013). Natronoarchaeum philippinense sp. nov., a haloarchaeon isolated from commercial solar salt. *Int. J. Syst. Evol. Microbiol.* 63, 920–924. doi:10.1099/ijs.0.042549-0.

Shimoshige, H., Yamada, T., Minegishi, H., Echigo, A., Shimane, Y., Kamekura, M., et al. (2013). Halobaculum magnesiiphilum sp. nov., a magnesium-dependent haloarchaeon isolated from commercial salt. *Int. J. Syst. Evol. Microbiol.* 63, 861–866. doi:10.1099/ijs.0.037432-0.

Song, H. S., Cha, I.-T., Rhee, J.-K., Yim, K. J., Kim, A. Y., Choi, J.-S., et al. (2016). Halostella salina gen. nov., sp. nov., an extremely halophilic archaeon isolated from solar salt. *Int. J. Syst. Evol. Microbiol.* 66, 2740–2746. doi:10.1099/ijsem.0.001118.

Song, H. S., Cha, I. T., Yim, K. J., Lee, H. W., Hyun, D. W., Lee, S. J., et al. (2014). Halapricum salinum gen. nov., sp. nov., an extremely halophilic archaeon isolated from non-purified solar salt. *Antonie van Leeuwenhoek, Int. J. Gen. Mol. Microbiol.* 105, 979–986. doi:10.1007/s10482-014-0156-x.

Sorokin, D. Y., Abbas, B., Merkel, A. Y., Rijpstra, W. I. C., Sinninghe Damsté, J. S., Sukhacheva, M. V., et al. (2015). Methanosalsum natronophilum sp. Nov., and methanocalculus alkaliphilus sp. nov., haloalkaliphilic methanogens from hypersaline Soda Lakes. *Int. J. Syst. Evol. Microbiol.* 65, 3739–3745. doi:10.1099/ijsem.0.000488.

Sorokin, D. Y., Khijniak, T. V., Kostrikina, N. A., Elcheninov, A. G., Toshchakov, S. V., Bale, N. J., et al. (2018a). Natronobiforma cellulositropha gen. nov., sp. nov., a novel haloalkaliphilic member of the family Natrialbaceae (class Halobacteria) from hypersaline alkaline lakes. *Syst. Appl. Microbiol.* 41, 355–362. doi:10.1016/j.syapm.2018.04.002.

Sorokin, D. Y., Kublanov, I. V., Yakimov, M. M., Rijpstra, W. I. C., and Sinninghe Damsté, J. S. (2016). Halanaeroarchaeum sulfurireducens gen. nov., sp. nov., the first obligately anaerobic sulfur-respiring haloarchaeon, isolated from a hypersaline lake. *Int. J. Syst. Evol. Microbiol.* 66, 2377–2381. doi:10.1099/ijsem.0.001041.

Sorokin, D. Y., Merkel, A. Y., Abbas, B., Makarova, K. S., Rijpstra, W. I. C., Koenen, M., et al. (2018b). Methanonatronarchaeum thermophilum gen. nov., sp. nov. and “Candidatus Methanohalarchaeum thermophilum”, extremely halo(natrono)philic methyl-reducing methanogens from hypersaline lakes comprising a new euryarchaeal class Methanonatronarchaeia classis nov. *Int. J. Syst. Evol. Microbiol.* 68, 2199–2208. doi:10.1099/ijsem.0.002810.

Sorokin, Di. Y., Messina, E., Smedile, F., Roman, P., Damsté, J. S. S., Ciordia, S., et al. (2017). Discovery of anaerobic lithoheterotrophic haloarchaea, ubiquitous in hypersaline habitats. *ISME*

J. 11, 1245–1260. doi:10.1038/ismej.2016.203.

Sowers, K. R., and Ferry, J. G. (1983). Isolation and Characterization of a Methylotrophic Marine Methanogen, Methanococcoides-Methylutens Gen-Nov, Sp-Nov. *Appl. Environ. Microbiol.* 45, 684–690.

Sprott, G. D., Agnew, B. J., and Patel, G. B. (1997). Structural features of ether lipids in the archaeobacterial thermophiles Pyrococcus furiosus, Methanopyrus kandleri, Methanothermus fervidus and Sulfolobus acidocaldarius. *Can. J. Microbiol.* 43, 467–476. doi:10.1139/m97-066.

Sprott, G. D., Brisson, J. R., Dicaire, C. J., Pelletier, A. K., Deschatelets, L. A., Krishnan, L., et al. (1999). A structural comparison of the total polar lipids from the human archaea Methanobrevibacter smithii and Methanosphaera stadtmanae and its relevance to the adjuvant activities of their liposomes. *Biochim. Biophys. Acta - Mol. Cell Biol. Lipids* 1440, 275–288. doi:10.1016/S1388-1981(99)00130-4.

Sprott, G. D., Dicaire, C. J., and Patel, G. B. (1994). The ether lipids of Methanosarcina mazei and other Methanosarcina species, compared by fast atom bombardment mass spectrometry. *Can. J. Microbiol.* 40, 837–843. doi:10.1139/m94-133.

Sprott, G. D., Larocque, S., Cadotte, N., Dicaire, C. J., McGee, M., and Brisson, J. R. (2003). Novel polar lipids of halophilic eubacterium Planococcus H8 and archaeon Haloferax volcanii. *Biochim. Biophys. Acta - Mol. Cell Biol. Lipids* 1633, 179–188. doi:10.1016/j.bbalip.2003.08.001.

St. John, E., Liu, Y., Podar, M., Stott, M. B., Meneghin, J., Chen, Z., et al. (2019). A new symbiotic nanoarchaeote (Candidatus Nanoclepta minutus) and its host (Zestosphaera tikiterensis gen. nov., sp. nov.) from a New Zealand hot spring. *Syst. Appl. Microbiol.* 42, 94–106. doi:10.1016/j.syapm.2018.08.005.

Stan-Lotter, H., Pfaffenhuemer, M., Legat, A., Busse, H. J., Radax, C., and Gruber, C. (2002). Halococcus dombrowskii sp. nov., an archaeal isolate from a Permian alpine salt deposit. *Int. J. Syst. Evol. Microbiol.* 52, 1807–1814. doi:10.1099/ijs.0.02278-0.

Sturt, H. F., Summons, R. E., Smith, K., Elvert, M., and Hinrichs, K. U. (2004). Intact polar membrane lipids in prokaryotes and sediments deciphered by high-performance liquid chromatography/electrospray ionization multistage mass spectrometry - New biomarkers for biogeochemistry and microbial ecology. *Rapid Commun. Mass Spectrom.* 18, 617–628. doi:10.1002/rcm.1378.

Sugai, A., Uda, I., Itoh, Y. H., and Itoh, T. (2004). The core lipid composition of the 17 strains of Thermococcales. *J. Oleo Sci.* 53, 41–44.

Takai, K., Nealson, K. H., and Horikoshi, K. (2004). Methanotorris formicicus sp. nov., a novel extremely thermophilic, methane-producing archaeon isolated from a black smoker chimney in the Central Indian Ridge. *Int. J. Syst. Evol. Microbiol.* 54, 1095–1100. doi:10.1099/ijs.0.02887-0.

Takai, K., Sugai, A., Itoh, T., and Horikoshi, K. (2000). Palaeococcus ferrophilus gen. nov., sp. nov., a barophilic, hyperthermophilic archaeon from a deep-sea hydrothermal vent chimney. *Int. J.*

Syst. Evol. Microbiol. 50, 489–500. doi:10.1099/00207713-50-2-489.

Takashina, T., Hamamoto, T., Otozai, K., Grant, W. D., and Horikoshi, K. (1990). Haloarcula japonica sp. nov., a New Triangular Halophilic Archaebacterium. *Syst. Appl. Microbiol.* 13, 177–181. doi:10.1016/S0723-2020(11)80165-7.

Takayanagi, S., Kawasaki, H., Sugimori, K., Yamada, T., Sugai, A., Ito, T., et al. (1996). Sulfolobus hakonensis sp. nov., a novel species of acidothermophilic archaeon. *Int. J. Syst. Bacteriol.* 46, 377–382. doi:10.1099/00207713-46-2-377.

Tapingkae, W., Tanasupawat, S., Itoh, T., Parkin, K. L., Benjakul, S., Visessanguan, W., et al. (2008). Natrinema gari sp. nov., a halophilic archaeon isolated from fish sauce in Thailand. *Int. J. Syst. Evol. Microbiol.* 58, 2378–2383. doi:10.1099/ijs.0.65644-0.

Thurl, S., and Schäfer, W. (1988). Lipids from the sulphur-dependent archaebacterimn Thermoproteus tenax. *Biochim. Biophys. Acta* 961, 253–261.

Tindall, B. J. (1990). Lipid composition of Halobacterium lacusprofundi. *FEMS Microbiol. Lett.* 66, 199–202. doi:10.1016/0378-1097(90)90282-U.

Tindall, B. J., Ross, H. N. M., and Grant, W. D. (1984). Natronobacterium gen. nov. and Natronococcus gen. nov., Two New Genera of Haloalkaliphilic Archaebacteria. *Syst. Appl. Microbiol.* 5, 41–57. doi:10.1016/S0723-2020(84)80050-8.

Tindall, B. J., Tomlinson, G. A., and Hochstein, L. I. (1989). Transfer of Halobacterium denitrificans (Tomlinson, Jahnke, and Hochstein) to the Genus Haloferax as Haloferax denitrificans comb. nov. *Int. J. Syst. Bacteriol.* 39, 359–360. doi:10.1099/00207713-39-3-359.

Torreblanca, M., Rodriguez-Valera, F., Juez, G., Ventosa, A., Kamekura, M., and Kates, M. (1986). Classification of Non-alkaliphilic Halobacteria Based on Numerical Taxonomy and Polar Lipid Composition, and Description of Haloarcula gen. nov. and Haloferax gen. nov. *Syst. Appl. Microbiol.* 8, 89–99. doi:10.1016/S0723-2020(86)80155-2.

Trigui, H., Masmoudi, S., Brochier-Armanet, C., Maalej, S., and Dukan, S. (2011). Characterization of halorubrum sfaxense sp. nov., a new halophilic archaeon isolated from the solar saltern of Sfax in Tunisia. *Int. J. Microbiol.* 2011. doi:10.1155/2011/240191.

Trincone, A., Lanzotti, V., Nicolaus, B., Zillig, W., De Rosa, M., and Gambacorta, A. (1989). Comparative lipid composition of aerobically and anaerobically grown Desulfurolobus ambivalens, an autotrophic thermophilic archaeobacterium. *Lipids* 135, 2751–2757.

Trincone, A., Nicolaus, B., Palmieri, G., De Rosa, M., Huber, R., Huber, G., et al. (1992). Distribution of complex and core lipids within new hyperthermophilic members of the Archaea domain. *Syst. Appl. Microbiol.* 15, 11–17. doi:10.1016/S0723-2020(11)80130-X.

Tsuboi, K., Sakai, H. D., Nur, N., Stedman, K. M., Kurosawa, N., and Suwanto, A. (2018). Sulfurisphaera javensis sp. nov., a hyperthermophilic and acidophilic archaeon isolated from indonesian hot spring, and reclassification of Sulfolobus tokodaii Suzuki et al. 2002 as Sulfurisphaera tokodaii comb. nov. *Int. J. Syst. Evol. Microbiol.* 68, 1907–1913. doi:10.1099/ijsem.0.002765.

Tsunogai, U., Ishibashi, J., Wakita, H., Gamo, T., Watanabe, K., Kajimura, T., et al. (1994). Peculiar features of Suiyo Seamount hydrothermal fluids, Izu-Bonin Arc: Differences from subaerial volcanism. *Earth Planet. Sci. Lett.* 126, 289–301. doi:10.1016/0012-821X(94)90113-9.

Uda, I., Sugai, A., Itoh, Y. H., and Itoh, T. (2004). Variation in molecular species of core lipids from the order Thermoplasmales strains depends on the growth temperature. *J. Oleo Sci.* 53, 399–404. doi:10.5650/jos.53.399.

Ventosa, A., Gutierrez, M. C., Kamekura, M., and Dyall-Smith, M. L. (1999). Proposal to transfer Halococcus turkmenicus, Halobacterium trapanicum JCM 9743 and strain GSL-11 to Haloterrigena turkmenica gen. nov., comb. nov. *Int. J. Syst. Bacteriol.* 49, 131–136. doi:10.1099/00207713-49-1-131.

Ventosa, A., Gutiérrez, M. C., Kamekura, M., Zvyagintseva, I. S., and Oren, A. (2004). Taxonomic study of Halorubrum distributum and proposal of Halorubrum terrestre sp. nov. *Int. J. Syst. Evol. Microbiol.* 54, 389–392. doi:10.1099/ijs.0.02621-0.

Völkl, P., Huber, R., Drobner, E., Rachel, R., Burggraf, S., Trincone, A., et al. (1993). Pyrobaculum aerophilum sp. nov., a novel nitrate-reducing hyperthermophilic archaeum. *Appl. Environ. Microbiol.* 59, 2918–26. Available at: http://www.ncbi.nlm.nih.gov/pubmed/7692819%0Ahttp://www.pubmedcentral.nih.gov/articlerender.fcgi?artid=PMC182387.

Von Damm, K. L. (1995). Controls on the chemistry and temporal variability of seafloor hydrothermal fluids. *Geophys. Monogr. Ser.* 91, 222–247. doi:10.1029/GM091p0222.

Vreeland, R., Straight, S., Krammes, J., Dougherty, K., Rosenzweig, W., and Kamekura, M. (2002). Halosimplex carlsbadense gen. nov., sp. nov., a unique halophilic archaeon, with three 16S rRNA genes, that grows only in defined medium with glycerol and acetate or pyruvate. *Extremophiles* 6, 445–452. doi:10.1007/s00792-002-0278-3.

Wagner, D., Schirmack, J., Ganzert, L., Morozova, D., and Mangelsdorf, K. (2013). Methanosarcina soligelidi sp. nov., a desiccation- and freeze-thaw-resistant methanogenic archaeon from a Siberian permafrost-affected soil. *Int. J. Syst. Evol. Microbiol.* 63, 2986–2991. doi:10.1099/ijs.0.046565-0.

Wainø, M., Tindall, B. J., and Ingvorsen, K. (2000). Halorhabdus utahensis gen. nov., sp. nov., an aerobic, extremely halophilic member of the Archaea from Great Salt Lake, Utah. *Int. J. Syst. Evol. Microbiol.* 50, 183–190. doi:10.1099/00207713-50-1-183.

Wang, Q. F., Li, W., Yang, H., Liu, Y. L., Cao, H. H., Dornmayr-Pfaffenhuemer, M., et al. (2007). Halococcus qingdaonensis sp. nov., a halophilic archaeon isolated from a crude sea-salt sample. *Int. J. Syst. Evol. Microbiol.* 57, 600–604. doi:10.1099/ijs.0.64673-0.

Wang, S., Yang, Q., Liu, Z. H., Sun, L., Wei, D., Zhang, J. Z., et al. (2010). Haloterrigena daqingensis sp. nov., an extremely haloalkaliphilic archaeon isolated from a saline-alkaline soil. *Int. J. Syst. Evol. Microbiol.* 60, 2267–2271. doi:10.1099/ijs.0.013995-0.

Wang, Z., Xu, J.-Q., Xu, W.-M., Li, Y., Zhou, Y., Lü, Z.-Z., et al. (2016). Salinigranum salinum sp. nov., isolated from a marine solar saltern. *Int. J. Syst. Evol. Microbiol.* 66, 3017–3021.

doi:10.1099/ijsem.0.001138.

Webber, A., Roberts, S., Murton, B., Mills, R., and Hodgkinson, M. (2017). The formation of gold-rich seafloor sulfide deposits: Evidence from the Beebe hydrothermal vent field, Cayman Trough. *Geochemistry, Geophys. Geosystems* 18, 2011–2027. doi:10.1002/2017GC006922.Received.

Wenzhöfer, F., Holby, O., Glud, R. N., Nielsen, H. K., and Gundersen, J. K. (2000). In situ microsensor studies of a shallow water hydrothermal vent at. *Milos, Greece. Mar. Chem.* 69, 43–54.

Xin, H., Itoh, T., Zhou, P., Suzuki, K., Kamekura, M., and Nakase, T. (2000). Natrinema versiforme sp. nov., an extremely halophilic archaeon from Aibi salt lake, Xinjiang, China. *Int. J. Syst. Evol. Microbiol.* 50, 1297–1303. doi:10.1099/00207713-50-3-1297.

Xin, H., Itoh, T., Zhou, P., Suzuki, K., and Nakase, T. (2001). Natronobacterium nitratireducens sp. nov., a haloalkaliphilic archaeon isolated from a soda lake in China. *Int. J. Microbiol.* 51, 1825–1829. doi:10.1099/00207713-51-5-1825.

Xu, J. Q., Xu, W. M., Li, Y., Zhou, Y., Lü, Z. Z., Hou, J., et al. (2016a). Halorussus salinus sp. nov., isolated from a marine solar saltern. *Arch. Microbiol.* 198, 957–961. doi:10.1007/s00203-016-1253-1.

Xu, Q., Cui, H.-L., and Meng, F. (2018). Haloprofundus halophilus sp. nov., isolated from the saline soil of Tarim Basin. *Antonie Van Leeuwenhoek* 6. doi:10.1007/s10482-018-1186-6.

Xu, W.-D., Zhang, W.-J., Han, D., Cui, H.-L., and Yang, K. (2015). Halorussus ruber sp. nov., isolated from an inland salt lake of China. *Arch. Microbiol.* 197, 91–95. doi:10.1007/s00203-014-1058-z.

Xu, W.-M., Xu, J.-Q., Zhou, Y., Li, Y., Lü, Z.-Z., Hou, J., et al. (2016b). Halomarina salina sp. nov., isolated from a marine solar saltern. *Antonie Van Leeuwenhoek* 109, 1121–1126. doi:10.1007/s10482-016-0714-5.

Xu, X. W., Liu, S. J., Tohty, D., Oren, A., Wu, M., and Zhou, P. J. (2005a). Haloterrigena saccharevitans sp. nov., an extremely halophilic archaeon from Xin-Jiang, China. *Int. J. Syst. Evol. Microbiol.* 55, 2539–2542. doi:10.1099/ijs.0.63761-0.

Xu, X. W., Ren, P. G., Liu, S. J., Wu, M., and Zhou, P. J. (2005b). Natrinema altunense sp. nov., an extremely halophilic archaeon isolated from a salt lake in Altun Mountain in Xinjiang, China. *Int. J. Syst. Evol. Microbiol.* 55, 1311–1314. doi:10.1099/ijs.0.63622-0.

Xu, X. W., Wu, M., Zhou, P. J., and Liu, S. J. (2005c). Halobiforma lacisalsi sp. nov., isolated from a salt lake in China. *Int. J. Syst. Evol. Microbiol.* 55, 1949–1952. doi:10.1099/ijs.0.63742-0.

Xu, X. W., Wu, Y. H., Wang, C. S., Oren, A., Zhou, P. J., and Wu, M. (2007a). Haloferax larsenii sp. nov., an extremely halophilic archaeon from a solar saltern. *Int. J. Syst. Evol. Microbiol.* 57, 717–720. doi:10.1099/ijs.0.64573-0.

Xu, X. W., Wu, Y. H., Zhang, H. Bin, and Wu, M. (2007b). Halorubrum arcis sp. nov., an extremely

halophilic archaeon isolated from a saline lake on the Qinghai-Tibet Plateau, China. *Int. J. Syst. Evol. Microbiol.* 57, 1069–1072. doi:10.1099/ijs.0.64921-0.

Xu, Y., Wang, Z., Xue, Y., Zhou, P., Ma, Y., Ventosa, A., et al. (2001). Natrialba hulunbeirensis sp. nov. and Natrialba chahannaoensis sp. nov., novel haloalkaliphilic archaea from soda lakes in Inner Mongolia Autonomous Region, China. *Int. J. Syst. Evol. Microbiol.* 51, 1693–1698. doi:10.1099/00207713-51-5-1693.

Xu, Y., Zhou, P., and Tian, X. (1999). Characterization of two novel haloalkaliphilic archaea Natronorubrum bangense gen. nov., sp. nov. and Natronorubrum tibetense gen. nov., sp. nov. *Int. J. Syst. Bacteriol.* 49, 261–266. doi:10.1099/00207713-49-1-261.

Xue, Y., Fan, H., Ventosa, A., Grant, W. D., Jones, B. E., Cowan, D. A., et al. (2005). Halalkalicoccus tibetensis gen. nov., sp. nov., representing a novel genus of haloalkaliphilic archaea. *Int. J. Syst. Evol. Microbiol.* 55, 2501–2505. doi:10.1099/ijs.0.63916-0.

Yamauchi, Y., Minegishi, H., Echigo, A., Shimane, Y., Kamekura, M., Itoh, T., et al. (2013). Halarchaeum rubridurum sp. nov., a moderately acidophilic haloarchaeon isolated from commercial sea salt samples. *Int. J. Syst. Evol. Microbiol.* 63, 3143–3147. doi:10.1099/ijs.0.049262-0.

Yang, X., and Cui, H. L. (2012). Halomicrobium zhouii sp. nov., a halophilic archaeon from a marine solar saltern. *Int. J. Syst. Evol. Microbiol.* 62, 1235–1240. doi:10.1099/ijs.0.031989-0.

Yang, Y., Cui, H. L., Zhou, P. J., and Liu, S. J. (2006). Halobacterium jilantaiense sp. nov., a halophilic archaeon isolated from a saline lake in Inner Mongolia, China. *Int. J. Syst. Evol. Microbiol.* 56, 2353–2355. doi:10.1099/ijs.0.64437-0.

Yang, Y., Cui, H. L., Zhou, P. J., and Liu, S. J. (2007). Haloarcula amylolytica sp. nov., an extremely halophilic archaeon isolated from Aibi salt lake in Xin-Jiang, China. *Int. J. Syst. Evol. Microbiol.* 57, 103–106. doi:10.1099/ijs.0.64647-0.

Yim, K. J., Cha, I. T., Lee, H. W., Song, H. S., Kim, K. N., Lee, S. J., et al. (2014a). Halorubrum halophilum sp. nov., an extremely halophilic archaeon isolated from a salt-fermented seafood. *Antonie van Leeuwenhoek, Int. J. Gen. Mol. Microbiol.* 105, 603–612. doi:10.1007/s10482-014-0115-6.

Yim, K. J., Cha, I. T., Whon, T. W., Lee, H. W., Song, H. S., Kim, K. N., et al. (2014b). Halococcus sediminicola sp. Nov., an extremely halophilic archaeon isolated from a marine sediment. *Antonie van Leeuwenhoek, Int. J. Gen. Mol. Microbiol.* 105, 73–79. doi:10.1007/s10482-013-0054-7.

Yin, S., Wang, Z., Xu, J. Q., Xu, W. M., Yuan, P. P., and Cui, H. L. (2015). Halorubrum rutilum sp. nov. isolated from a marine solar saltern. *Arch. Microbiol.* 197, 1159–1164. doi:10.1007/s00203-015-1159-3.

Yin, X.-Q., Liu, B.-B., Chu, X., Salam, N., Li, X., Yang, Z.-W., et al. (2017). Saliphagus infecundisoli gen. nov., sp. nov., an extremely halophilic archaeon isolated from a saline soil. *Int. J. Syst. Evol. Microbiol.* 67, 4154–4160. doi:10.1099/ijsem.0.002270.

Yoshinaga, M. Y., Gagen, E. J., Wörmer, L., Broda, N. K., Meador, T. B., Wendt, J., et al. (2015). Methanothermobacter thermautotrophicus modulates its membrane lipids in response to hydrogen and nutrient availability. *Front. Microbiol.* 6, 1–9. doi:10.3389/fmicb.2015.00005.

Yuan, P.-P., Sun, X.-J., Liang, X., Chen, X.-J., Han, D., Zhang, W.-J., et al. (2015a). Haloarchaeobius amylolyticus sp. nov., isolated from a marine solar saltern. *Arch. Microbiol.* 197, 949–953. doi:10.1007/s00203-015-1129-9.

Yuan, P. P., Xu, J. Q., Xu, W. M., Wang, Z., Yin, S., Han, D., et al. (2015b). Halosimplex litoreum sp. nov., isolated from a marine solar saltern. *Antonie van Leeuwenhoek, Int. J. Gen. Mol. Microbiol.* 108, 483–489. doi:10.1007/s10482-015-0501-8.

Yuan, P. P., Ye, W. T., Pan, J. X., Han, D., Zhang, W. J., and Cui, H. L. (2015c). Halorussus amylolyticus sp. Nov., isolated from an inland Salt Lake. *Int. J. Syst. Evol. Microbiol.* 65, 3734–3738. doi:10.1099/ijsem.0.000487.

Yuan, P. P., Yin, S., Han, D., Zhang, W. J., and Cui, H. L. (2015d). Halorientalis brevis sp. nov., Isolated from an Inland Salt Lake of China. *Curr. Microbiol.* 71, 382–386. doi:10.1007/s00284-015-0861-3.

Yuan, P. P., Zhang, W. J., Han, D., and Cui, H. L. (2015e). Haloarchaeobius salinus sp. nov., isolated from an inland salt lake, and emended description of the genus Haloarchaeobius. *Int. J. Syst. Evol. Microbiol.* 65, 910–914. doi:10.1099/ijs.0.000038.

Zellner, G., Boone, D. R., Keswani, J., Whitman, W. B., Woese, C. R., Hagelstein, A., et al. (1999). Reclassification of Methanogenium tationis and Methanogenium liminatans as Methanofollis tationis gen. nov., comb. nov. and Methanofollis liminatans comb. nov. and description of a new strain of Methanofollis liminatans. *Int. J. Syst. Bacteriol.* 49, 247–255. doi:10.1099/00207713-49-1-247.

Zellner, G., Messner, P., Kneifel, H., Tindall, B. J., Winter, J., and Stackebrandt, E. (1989a). Methanolacinia gen. nov., incorporating Methanomicrobium paynteri as Methanolacinia paynteri comb. nov. *J. Gen. Appl. Microbiol.* 35, 185–202. doi:10.2323/jgam.35.185.

Zellner, G., Stackebrandt, E., Messner, P., Tindall, B. J., Maeario, E. C. De, Kneifel, H., et al. (1989b). Methanocorpusculaceae fam. nov., represented by Methanocorpusculum parvum, Methanocorpusculum sinense spec. nov. and Methanocorpusculum bavaricum spec. nov. *Arch. Microbiol.* 151, 381–390.

Zeng, Z., Liu, X.-L., Wei, J. H., Summons, R. E., and Welander, P. V. (2018). Calditol-linked membrane lipids are required for acid tolerance in Sulfolobus acidocaldarius. *Proc. Natl. Acad. Sci.* 115, 12932–12937. doi:10.1073/pnas.1814048115.

Zhang, G., Gu, J., Zhang, R., Rashid, M., Haroon, M. F., Xun, W., et al. (2017). Haloprofundus marisrubri gen. nov., sp. nov., an extremely halophilic archaeon isolated from a brine–seawater interface. *Int. J. Syst. Evol. Microbiol.* 67, 9–16. doi:10.1099/ijsem.0.001559.

Zhang, W.-J., and Cui, H.-L. (2014a). Halorubrum salinum sp. nov., isolated from a marine solar saltern. *Arch. Microbiol.* 196, 395–400. doi:10.1007/s00203-014-0975-1.

Zhang, W. J., and Cui, H. L. (2014b). Haloarchaeobius litoreus sp. nov., isolated from a marine solar saltern. *Antonie van Leeuwenhoek, Int. J. Gen. Mol. Microbiol.* 105, 1085–1090. doi:10.1007/s10482-014-0166-8.

Zhang, W. J., and Cui, H. L. (2014c). Halomicroarcula limicola sp. nov., isolated from a marine solar saltern, and emended description of the genus Halomicroarcula. *Int. J. Syst. Evol. Microbiol.* 64, 1747–1751. doi:10.1099/ijs.0.062455-0.

Zhang, W. J., and Cui, H. L. (2015). Halomicroarcula salina sp. nov., isolated from a marine solar saltern. *Int. J. Syst. Evol. Microbiol.* 65, 1628–1633. doi:10.1099/ijs.0.000150.

Zhang, W. J., Han, D., Qiu, X. X., Zhao, M. L., Mou, Y. Z., Cui, H. L., et al. (2013a). Halobellus rarus sp. nov., a halophilic archaeon from an inland salt lake of China. *Antonie van Leeuwenhoek, Int. J. Gen. Mol. Microbiol.* 104, 377–384. doi:10.1007/s10482-013-9960-y.

Zhang, W. Y., Huo, Y. Y., Zhang, X. Q., Zhu, X. F., and Wu, M. (2013b). Halolamina salifodinae sp. nov. and Halolamina salina sp. nov., two extremely halophilic archaea isolated from a salt mine. *Int. J. Syst. Evol. Microbiol.* 63, 4380–4385. doi:10.1099/ijs.0.050864-0.

Zhang, W. Y., Meng, Y., Zhu, X. F., and Wu, M. (2013c). Halopiger salifodinae sp. nov., an extremely halophilic archaeon isolated from a salt mine. *Int. J. Syst. Evol. Microbiol.* 63, 3563–3567. doi:10.1099/ijs.0.050971-0.

Zhang, X., Zhang, W. Y., Shen, A. H., Huo, Y. Y., Zhu, X. F., and Wu, M. (2013d). Halopelagius longus sp. nov., a member of the family Halobacteriaceae isolated from a salt mine, and emended description of the genus Halopelagius. *Int. J. Syst. Evol. Microbiol.* 63, 3585–3590. doi:10.1099/ijs.0.051375-0.

Zhao, B., Hu, Q., Guo, X., Liao, Z., Sarmiento, F., Mesbah, N. M., et al. (2018). Natronolimnobius aegyptiacus sp. nov., an extremely halophilic alkalithermophilic archaeon isolated from the athalassohaline Wadi An Natrun, Egypt. *Int. J. Syst. Evol. Microbiol.* 68, 498–506. doi:10.1099/ijsem.0.002524.

Zhao, M.-L., Qiu, X.-X., Zhang, W.-J., Han, D., Cui, H.-L., and Li, Z.-R. (2014). Halobellus litoreus sp. nov., a Halophilic Archaeon Isolated from a Chinese Marine Solar Saltern. *Curr. Microbiol.* 68, 156–160. doi:10.1007/s00284-013-0457-8.

Zhou, Y., Li, Y., Lü, Z. Z., and Cui, H. L. (2017). Halomarina rubra sp. nov., isolated from a marine solar saltern. *Arch. Microbiol.* 199, 1431–1435. doi:10.1007/s00203-017-1420-z.

Zillig, W., Gierl, A., Schreiber, G., Wunderl, S., Janekovic, D., Stetter, K. O., et al. (1983). The archaebacterium Thermofilum pendens represents, a novel genus of the thermophilic, anaerobic sulfur respiring Thermoproteales. *Syst. Appl. Microbiol.* 4, 79–87. doi:10.1016/s0723-2020(83)80035-6.

Zillig, W., Holz, I., Janekovic, D., Klenk, H. P., Imsel, E., Trent, J., et al. (1990). Hyperthermus butylicus, a hyperthermophilic sulfur-reducing archaebacterium that ferments peptides. *J. Bacteriol.* 172, 3959–3965. doi:10.1128/jb.172.7.3959-3965.1990.
